# High bacterial diversity and siderophore-producing bacteria collectively suppress *Fusarium oxysporum* in maize/faba bean intercropping

**DOI:** 10.3389/fmicb.2022.972587

**Published:** 2022-08-05

**Authors:** Xinzhan Sun, Chaochun Zhang, Shuikuan Bei, Guangzhou Wang, Stefan Geisen, Laurent Bedoussac, Peter Christie, Junling Zhang

**Affiliations:** ^1^College of Resources and Environmental Sciences, National Academy of Agriculture Green Development, Key Laboratory of Plant-Soil Interactions, Ministry of Education, China Agricultural University, Beijing, China; ^2^Laboratory of Nematology, Wageningen University, Wageningen, Netherlands; ^3^AGIR, University of Toulouse, ENSFEA, INRAE, Castanet-Tolosan, France

**Keywords:** intercropping, soil-borne pathogen, microbial networks, siderophore-producing bacteria, bacterial community

## Abstract

Beyond interacting with neighboring plants, crop performance is affected by the microbiome that includes pathogens and mutualists. While the importance of plant–plant interactions in explaining overyielding in intercropping is well known, the role of the microbiome, in particular how the presence of microbes from heterospecific crop species inhibit pathogens of the focal plants in affecting yield remains hardly explored. Here we performed both field samplings and pot experiments to investigate the microbial interactions in the maize/faba bean intercropping system, with the focus on the inhibition of *Fusarium oxysporum* in faba bean plants. Long-term field measurements show that maize/faba bean intercropping increased crop yield, reduced the gene copies of *F. oxysporum* by 30–84% and increased bacterial richness and Shannon index compared to monocropping. Bacterial networks in intercropping were more stable with more hub nodes than the respective monocultures. Furthermore, the observed changes of whole microbial communities were aligned with differences in the number of siderophore-producing rhizobacteria in maize and pathogen abundances in faba bean. Maize possessed 71% more siderophore-producing rhizobacteria and 33% more synthetases genes abundance of nonribosomal peptides, especially pyochelin, relative to faba bean. This was further evidenced by the increased numbers of siderophore-producing bacteria and decreased gene copies of *F. oxysporum* in the rhizosphere of intercropped faba bean. Four bacteria (*Pseudomonas* spp. B004 and B021, *Bacillus* spp. B005 and B208) from 95 isolates antagonized *F. oxysporum* f. sp. *fabae*. In particular, B005, which represented a hub node in the networks, showed particularly high siderophore-producing capabilities. Intercropping increased overall bacterial diversity and network complexity and the abundance of siderophore-producing bacteria, leading to facilitated pathogen suppression and increased resistance of faba bean to *F. oxysporum*. This study has great agronomic implications as microorganisms might be specifically targeted to optimize intercropping practices in the future.

## Introduction

Food security is a major challenge with a growing population and increasing demand for healthy food (Tilman et al., 2011). However, global food production is subject to various threats, in which soil-borne pathogens account for approximately 20% of yield losses (Fisher et al., 2012; Savary et al., 2019). Intensive large-scale monocultures are prone to disease outbreaks, which cause yield losses (Zhao et al., 2017; Li et al., 2021a). Fungal disease can be effectively controlled by fungicide applications (Le Cointe et al., 2016), or alternatively through sustainable diversified cropping systems such as rotations and/or intercropping (Abawi and Widmer, 2000). In the latter case, cereal/legume intercropping systems are the most explored to give less reliance on fertilizer N and high-efficiency use of P (Fisher and Long, 1992; Hauggaard-Nielsen et al., 2009) due to complementarity in resource use and facilitation in N and P between the interspecific plant species involved (Li et al., 2007, 2016). Such facilitative effects are explained by the direct contribution of plant root traits such as root exudates, signals and root trait plasticity (Yu et al., 2021), or the enhanced beneficial microbial interactions such as nitrogen fixation (Zhang et al., 2010) and arbuscular mycorrhizal fungi (Gomez-Sagasti et al., 2021). However, it remains largely unexplored whether these facilitative effects are associated with disease suppression, e.g., microbes favoring one crop species reducing pathogen attack or herbivores on neighboring focal plant species.

A growing body of studies on the suppression of soil-borne disease in the intercropping focuses mostly on the dilution effect or the interactions between root exudates and pathogens, e.g., exudates from one crop inhibit the disease incidences of the neighboring plants (Boudreau, 2013; Yu et al., 2021). For example, phenolic acids, organic acids, amino acids, and sugars are known to decrease disease incidence (Lv et al., 2020), while cinnamic acid increases disease incidence (Guo et al., 2020). Aside from these mechanisms, plants are tightly linked to species-specific microbiomes that themselves directly interact with soil-borne pathogens (Pang et al., 2022). Plant core microbiome (Toju et al., 2018) and keystone microbial taxa (Banerjee et al., 2018) associated plants promote plant growth and inhibit pathogen occurrence (Lemanceau et al., 2017), and soil biota has positive legacy effects on the growth and health of plants (Kuerban et al., 2022; Wang and Song, 2022). For example, the enrichment of specific microbial taxa such as *Pseudomonas* (Song et al., 2021) or *Xanthomonas*, *Stenotrophomonas*, and *Microbacterium* spp. (Berendsen et al., 2018) are associated with pathogen suppression. Recent studies also indicate that the microbial network of healthy peanut plants is more complex than that of peanut infected with the pathogen *Ralstonia* (Li et al., 2021a). Similar results have also been reported for tomato (Wei et al., 2018). In intercropping systems, the two co-cultivated crop species inevitably interact to affect soil microbial communities. It is thus envisaged that microbial communities in intercropping systems may enhance the overall ecological interactions in the rhizosphere microbiome to suppress soil-borne diseases, or specific bacterial taxa from one crop species may directly reduce the soil-borne pathogens of the neighboring plant species. However, so far there still lacks of direct evidence for this.

Both plants and pathogens require iron for normal growth and the capacity for siderophore-production is highly correlated with pathogen suppressiveness and plant health (Kramer et al., 2020). Cereals such as maize are strategy II plants that mobilize iron by releasing phytosiderophores (Marschner and Römheld, 1994). In contrast, legumes such as faba bean and chickpea acidify the rhizosphere by releasing protons and organic acids (Li et al., 2007; Wen et al., 2021), which may increase Fe availability but decrease siderophore production by bacteria. Most microbial siderophores are synthesized by nonribosomal peptide synthetase (NRPS)-dependent pathway (Kadi and Challis, 2009; Verbon et al., 2017), and act as microbicides to suppress plant pathogens (Guo et al., 2022), such as the pyochelin (De Vleesschauwer et al., 2006) and enterochelin (Lambrese et al., 2018). Hence, competition for iron between crop and rhizosphere microbiome may be an important mechanism explaining disease suppression, although other mechanisms such as antibiosis or activation of plant immunity are also involved (Li et al., 2021c). Here, the presence of maize plants may facilitate intercropped faba bean to outcompete pathogens. Faba bean is an economically important crop which is often attacked by soil-borne pathogens such as *F. oxysporum*. In the present study, we have conducted a series of experiments combining microbial community analysis with pot experiments using 16S rRNA high-throughput sequencing, shot-gun metagenomics, *in vitro* antagonistic test and inoculation bioassay analysis to unravel the impact of rhizosphere bacteria in maize/faba bean intercropping on Fusarium wilt disease (*F. oxysporum*) of faba bean. We hypothesized that: (1) rhizosphere bacterial diversity and network connections are enhanced in the intercropping, and the abundance of *F. oxysporum* declines; (2) bacteria in the maize rhizosphere may possess more siderophore-producing bacteria than those in the faba bean rhizosphere, which are involved in the suppression of *F. oxysporum* in the faba bean rhizosphere; and (3) bacterial isolates exhibiting strong antagonistic effects on *F. oxysporum* may possess high siderophore production capacity.

## Materials and methods

### Field experiment

**Figure 1:**
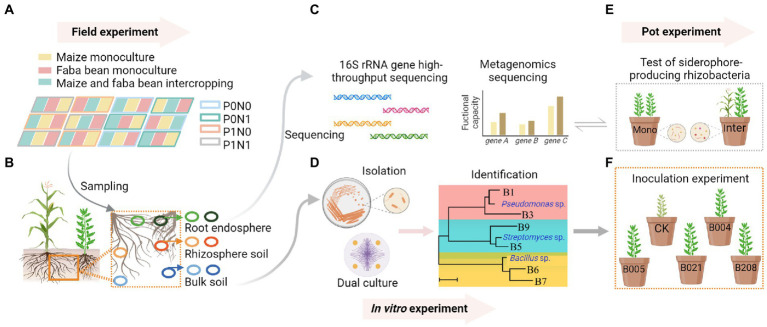
In the field experiment, the field plots comprised three planting patterns (monocultures of maize and faba bean, maize/faba bean intercropping) and four fertilization treatments P0N0: no fertilization; P0N1: sole N fertilization at 180 kg N ha^−1^ yr.^−1^ as urea; P1N0: sole P fertilization at 40 kg P ha^−1^ yr.^−1^ as superphosphate; P1N1: P and N fertilization **(A)**. Samples were collected from bulk soil (BS), rhizosphere soil (RS) and root endosphere (RE) of maize and faba bean **(B)**. DNA was extracted from soil and root samples for 16S rRNA gene high-throughput sequencing, and the siderophore-producing capacity of rhizosphere soil in maize and faba bean was analyzed by shotgun metagenomic sequencing **(C)**. Bacterial isolation, screening, identification and dual culture against *Fusarium oxysporum* were conducted *in vitro* **(D)**. Pot experiments compared the numbers of siderophore-producing rhizobacteria and the gene copies of *F. oxysporum* of faba bean between monoculture and intercropping **(E)**. An inoculation experiment tested the effects of selected isolates on disease suppression of faba bean **(F)**. Created with BioRender.com.

A long-term field experiment was established in 2009 at Quzhou County (36.93 N, 115.17E; 40 m above sea level) in Hebei Province, north China. The soil is a calcareous fluvisol with a pH of 7.3. The climate is typical monsoon with an annual mean precipitation of 556 mm and mean temperature of 13.1°C. The properties of the topsoil (0–20 cm depth) were: organic matter content 14.0 g kg^−1^, total nitrogen content 0.84 g kg^−1^, Olsen-P 12.6 mg kg^−1^, and exchangeable potassium 0.21 g kg^−1^ (Liao et al., 2020). The field experiment is a split-plot design with four fertilization treatments (Figure 1): (1) no fertilization (P0N0), (2) N fertilization alone (P0N1) at 180 kg N ha^−1^ yr.^−1^ as urea, (3) P fertilization alone (P1N0) at 40 kg P ha^−1^ yr.^−1^ as superphosphate, and (4) N and P fertilization (P1N1) at 180 kg N ha^−1^ yr.^−1^ and 40 kg P ha^−1^ yr.^−1^. The sub-plots comprise three planting patterns: (1) monocultured maize (*Zea mays* L. cv. Zhengdan 958), (2) monocultured faba bean (*Vicia faba* L. cv. Lincan 5), and (3) maize/faba bean intercropping. There are three replicate plots of each treatment, giving a total of 36 plots. Details of the agronomic management are presented in the Supplementary material.

### Crop harvest and intercropping overyielding calculation

From the crop yield monitoring area located in the central strip of each plot we collected all corncobs of two rows of maize with 10 adjacent plants in each row, or all pods of three rows of faba bean with 20 adjacent plants in each row. After the corncobs or faba bean pods were air-dried, the grains were separated manually and weighed for yield calculation. The partial land equivalent ratio (pLER) was calculated to explain intercropping advantage (Willey, 1979):


$$pLER=\frac{Y_{IN}\times RD}{Y_{IN}}$$

where Y_IN_ and Y_MO_ are the crop yields in intercropping and monoculture, respectively, and RD is the relative density of crops under intercropping relative to monoculture (57 and 43%, respectively, in maize and faba bean). The land equivalent ratio (LER) is the sum of the pLER values of maize and faba bean, and a value >1 indicates intercropping overyielding and an LER ≤ 1 indicates no overyielding.

### Soil sampling

Soil samples were collected from monoculture and intercropped maize and faba bean plots in June 2019, at the V10 stage of maize but the flowering stage of faba bean. The samples were separated into bulk soil (BS), rhizosphere soil (RS) and root endosphere (RE; Lundberg et al., 2012). In total, 144 samples were collected (3 compartments × 2 crop species × 2 planting patterns × 4 fertilization levels × 3 replicates). Details of sampling, determination of soil physicochemical properties, DNA extraction, real-time quantitative PCR, 16S rRNA gene amplification, high-throughput sequencing, bioinformatics analysis and co-occurrence network analysis are provided in the Supplementary material.

### Disease inhibition in intercropping (pot experiment)

A pot experiment was conducted to test whether intercropping affected the abundance of the Fusarium wilt pathogen, and the number of siderophore-producing rhizobacteria in the faba bean rhizosphere. A growth substrate comprising a mixture of 100 g field soils with 900 g sand was sterilized and faba bean was planted. Two seedlings of faba bean were grown in monoculture, and one seedling each of maize and faba bean in intercropping. Each treatment had three replicates. *F. oxysporum* f. sp. *fabae* (FOF) was isolated from diseased faba bean plants in the field. FOF spore suspension (1.0 × 10^5^ FOF spores g^−1^ substrate) was added 2 weeks after germination. Plants were grown in a chamber at 25°C and 60–80% relative humidity. Two weeks after FOF inoculation, faba bean was sampled for growth measurement, and rhizosphere soils were sampled for the determination of FOF abundance and the number of siderophore-producing bacteria.

### Counting siderophore-producing rhizobacteria and cultivation of *Fusarium oxysporum*

The siderophore-producing bacteria and *F. oxysporum* in the rhizosphere soils were sampled from the pot experiment. The bacteria and FOF were cultivated using specialized chrome azurol S medium (Schwynan and Neilands, 1987) and potato dextrose agar (PDA) medium, respectively. Soil suspension was processed by mixing 10 g fresh soil from zero P fertilizer treatment (field soils) with 90 ml sterile distilled water in 250-mL conical flasks to produce a 10^−1^ diluted suspension. The soil suspension was tenfold serially diluted to obtain dilutions of 10^−2^–10^−5^ in 10-mL centrifuge tubes. The suspensions in tubes were mixed by vortexing for 30 s. Suspensions (100 μl) were fully spread on PDA medium or chrome azurol S medium at pH 6.0. The plates were incubated at 30°C in the dark for 2 days followed by counting of colony-forming units (CFUs). The CFUs of the siderophore-producing bacteria was counted when the plaques larger than 1 mm in diameter or orange halo was produced on the azurol S medium. The CFUs of *F. oxysporum* were visually identified by morphological characteristics and fungal color.

### Metagenomic sequencing and NRPS gene annotation

Differences in potential functions between maize and faba bean microbiomes were compared using rhizosphere DNA samples of monocultured maize and faba bean grown in the fields in the zero P fertilizer treatment. DNA was fragmented to an average size of ~300 bp using a Covaris M220 focused ultrasonicator (Woburn, MA). Paired-end libraries were constructed using TruSeq DNA Sample Prep Kits (Illumina, San Diego, CA). Sequencing was conducted using the Illumina HiSeq 4,000 platform in 2 × 150 bp paired-end mode, resulting in a total dataset of 95.7 Gb. The raw sequence data were deposited in the Genome Sequence Archive under accession number CRA005329. The low-quality reads were filtered by Fastp and the high-quality reads were assembled with MEGAHIT (mink = 27, maxk = 141, and step = 20; Li et al., 2015). Contigs ≥300 bp were selected as the final assembly result for further gene prediction and annotation. The open reading frames (ORF) were predicted using MetaGene (Lv et al., 2014). The predicted ORFs with length ≥ 100 bp were retrieved and translated to amino acid sequences using the NCBI translation table. All sequences from gene sets with a 95% sequence identity (90% coverage) were clustered as the non-redundant gene catalog by CD-HIT (Fu et al., 2012). Finally, high-quality reads were compared with non-redundant gene sets (95% identity) using SOAAPaligner, and the abundance of genes in the corresponding samples was determined (Li et al., 2008). Representative sequences of the non-redundant gene catalog were compared (e-value threshold, 10^−5^) against databases COG (The Clusters of Orthologous Groups) and KEGG (the Kyoto Encyclopedia of Genes and Genomes) using BLASTP (Version 2.2.28+). The annotation results were extracted with KOBAS (v3.0.3) software (−t blastout:tab, −s ko; Xie et al., 2011). Nonribosomal peptide synthetase (NRPS) involved in the biosynthesis of hydroxamate-, catecholate-, and nitrogen heterocycle-based siderophores and peptide antibiotics were annotated as described previously (Kadi and Challis, 2009). Here, the genes involved in the biosynthesis of siderophore group nonribosomal peptides (KEGG pathway: ko01053, e.g., *pchD*, *mbtA*, *irp1*, *entB*, *mxcE*, *dhbB*, *cibB*) were selected (Satinsky et al., 2014).

### Cultivation of antagonistic bacteria

Bacterial strains were isolated from bulk soil across all treatments of the maize/faba bean intercropping system in the field experiment using the Luria-Bertani (LB) medium. Soil suspensions were processed by serial gradient dilution as described for the cultivation of siderophore-producing bacteria. The plates were incubated in the dark at 25°C for 2 days. Representative colonies were picked out from the plates and a repeat streaking procedure on new dishes with LB medium, incubated for another 2 days to obtain pure colonies. The bacterial isolates were stored in LB liquid medium containing 20% glycerol at −80°C. A total of 95 bacterial isolates were identified by 16S rRNA sequencing with the primers 27F/1492R following the protocol: initial denaturation at 98°C for 3 min, 30 cycles of 98°C for 10 s, 56°C for 10 s, and 72°C for 30 s, with a final extension at 72°C for 5 min (Heuer et al., 1997). The purified amplicons were sequenced by Tsingke Biotechnology Co., Ltd., Beijing, China, and their taxonomic identity was blasted at www.ncbi.nlm.nih.gov/blast/Blast.cgi.

The antagonistic activities of bacterial isolates against FOF were tested using the dual culture assays on PDA (Yin et al., 2021). Sole inoculation of FOF was used as the control. All culture plates were placed in the dark and incubated at 28°C for 5 days until the PDA medium in the controls was completely covered with FOF mycelia. The colony diameter was measured radially with a ruler. The inhibition rate was calculated as follows:


$$Inhibitionrate=\frac{\left(Control-Treatment\right)}{Control}\times 100\%$$

Four bacterial isolates (B004, B005, B021 and B208) showing relatively strong inhibition rates (30%) of radial fungal growth were selected for further experiments. A neighbor joining (NJ) phylogenetic tree was constructed by the sequences of four strongly antagonistic isolates and their reference sequences from GenBank: *Bacillus subtilis* (MH666097.1), *Bacillus megaterium* (MT487648.1), *Pseudomonas chlororaphis* (KU977134.1) and *P. chlororaphis* (HM241942.1), as well as all OTU representative references of *Bacillus* and *Pseudomonas*.

### FOF conidial germination and sporulation

Metabolites of the four strongly antagonistic bacterial isolates (B004, B005, B021 and B208) were used to test their antagonism against conidial germination and sporulation. First, the bacteria were separately cultured on the LB liquid medium at 28°C for 12 h when it reached the logarithmic growth stage. Then the bacteria solution was inoculated into the new LB medium at a dose of 1% and cultured at 28°C for 3 days. Afterwards, the liquid medium was centrifuged to obtain the supernatant. The supernatant was then filtered through 0.2-μm membrane filters to obtain sterile bacterial metabolites. Spore germination was evaluated by washing 7-day-old mycelia on PDA with sterile water and collecting the spores by filtering through two-layer lens papers. The spore suspensions were diluted to 1 × 10^7^ spores mL^−1^ and 1.0 ml of spore suspension and 9.0 ml sterilized bacterial metabolites (filtered through 0.2-μm membrane filters) were mixed. The tubes were incubated at 28°C for 12 h. A tube length of spore bud longer than the short radius of the spore was regarded as germination and the number of spores germinated were counted. Sporulation was assessed by cutting five 5-mm agar plugs from 7-day-old cultures and inoculating into 100 ml potato dextrose liquid medium containing 50% (v/v) bacterial metabolites, and incubated for 5 days at 28°C with constant shaking at 180 rpm. After inoculation, the culture broth was filtered through two layers of sterilized lens papers. The spore number was detected microscopically (YS100, Nikon, Tokyo, Japan) at 400 × magnification (Guo et al., 2020).

### Greenhouse plant assay (inoculation experiment)

Four bacterial isolates of B004, B005, B021, and B208 were used in a greenhouse bioassay experiment to test their inhibitory effects on FOF. Sand with diameter of 0.6–1.0 mm was autoclaved twice at 121°C for 30 min, and it was used as growth substrate. Faba bean seeds were surface – sterilized by immersion in 10% (v/v) hydrogen peroxide for 30 min, followed by three rinses with 300 ml sterile distilled water. Seeds were then placed in a rectangular dish (50 × 30 cm) containing sterile water and incubated at 25°C in the dark until the seeds germinated (after 60 h). Two faba bean seedlings were planted in each pot (6.0 × 5.0 × 7.5 cm) and one seedling was retained 14 days after planting. The four bacterial isolates were inoculated separately into the LB liquid medium and shaken at 30°C overnight. Cells were collected by centrifuging at 10000 rpm for 2 min and suspended in sterile distilled water to an optical density (OD_600_) value of 1.0. After the seedlings were thinned-out, 25 ml bacterial suspension were added near the plant roots, and control seedlings were supplied with an equal volume of sterile water. Two weeks later, FOF spore suspensions were added to all treatments (3.2 × 10^5^ FOF spores g^−1^ of sand). Each treatment had four replicates. Pots were arranged in a randomized complete block design in plastic plates and incubated in a greenhouse at 25–30°C (day) / 18–22°C (night) and 60–80% relative humidity. The seedlings were watered daily with ~25 ml per pot and harvested after 5 weeks. Disease indices were classified according to Lv et al. (2020) and recorded at harvest. The number of siderophore-producing rhizobacteria was counted on the chrome azurol S medium.

### Statistical analysis

Crop yields and *F. oxysporum* gene copies in the field experiment were analyzed using two-way analysis of variance (ANOVA) and mean values were compared by Student’s *t*-test or Duncan’s multiple range test using R version 4.1.0^1^.Spearman’scorrelation coefficients were calculated in R software between *F. oxysporum* gene copies and maize and faba bean yields. The inhibitory effects of *Bacillus* and *Pseudomonas* strains were tested by calculating the correlations between *F. oxysporum* gene copies and the relative abundance of *Bacillus* and *Pseudomonas*. Bacterial richness and Shannon index were calculated using “vegan” package. Two-way ANOVA was used to analyze alpha diversity differences between planting pattern and fertilization in each compartment of maize and faba bean. The microbial community structure was analyzed through principal coordinate analysis (PCoA) based on a Bray–Curtis distance matrix, and the coordinates were used to draw 2D graphical outputs (Chen et al., 2019). Permutational multivariate analysis of variance (PERMANOVA) was conducted to test the significance of planting pattern and fertilization using the *adonis* function in the “vegan” package with 999 permutations (Oksanen et al., 2007). In the pot experiment, Student’s t-test was used to assess the differences in the number of siderophore-producing rhizobacteria and the abundance of the pathogen *F. oxysporum* between planting patterns, as well as in the number of siderophore-producing rhizobacteria and the functional categories or genes of NRPS and iron metabolism between crop species in the metagenomic analysis. Duncan’s multiple range test was used in the inoculation experiment to test the antagonistic effects of the four isolates against *F. oxysporum*.

## Results

### Crop productivity and *Fusarium oxysporum* gene copies

**Figure 2:**
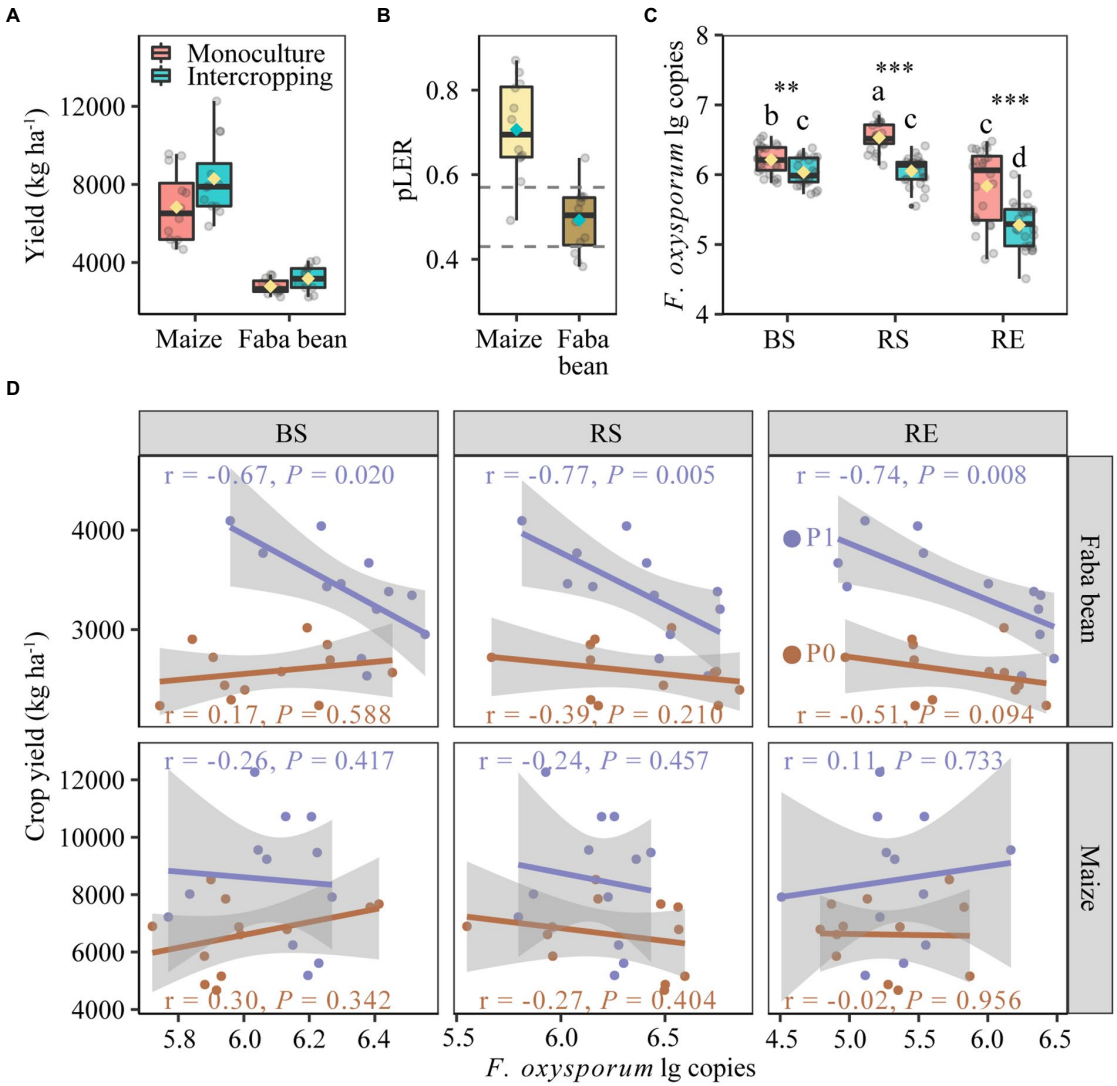
Yields of maize and faba in monoculture and intercropping **(A)**. Partial land equivalent ratio (pLER) of maize and faba bean in intercropping systems **(B)**. *Fusarium oxysporum* gene copies in bulk soil, rhizosphere and root endosphere of maize and faba bean **(C)**. Spearman correlations between yields of faba bean and maize with the abundance of *Fusarium oxysporum* in bulk soil, rhizosphere and root endosphere at zero P fertilization and 40 kg P ha^−1^ yr.^−1^ **(D)**. In each box plot the top and bottom of each box represent the 25th and 75th percentiles, the horizontal line inside each box represents the 50th percentile/median and the whiskers represent the range of the points excluding outliers. The diamonds of cyan and yellow are the average of the boxplot. In panel **(B)**. The values of dashed lines are the relative densities of intercropped maize (0.57) and intercropped faba bean (0.43) with respect to the respective monocultures. In panel **(C)**, bars with different letters indicate significant differences by Duncan’s multiple range test (*p* < 0.05). Monoculture, *n* = 24; intercropping, *n* = 24. BS, bulk soil; RS, rhizosphere soil; RE, root endosphere; P0, no P fertilization; P1, 40 kg P ha^−1^ yr.^−1^.

In the field experiment, intercropping increased the yields of maize and faba bean over monocropping by 21.3 and 14.4%, respectively (Figure 2A) and with an average LER of 1.2 across all treatments, indicating intercropping advantages (Figure 2B). Yields of maize and faba bean were significantly affected by fertilization and planting pattern (Supplementary Table S1), but pLER of faba bean was not significantly affected by fertilization treatment (*F* = 2.20, *p* = 0.17; Supplementary Table S2). Intercropping altered soil pH and N content while soil TC and AP contents were significantly affected by fertilization (Supplementary Table S3).

The *F. oxysporum* gene copies in different compartments of maize and faba bean were significantly affected by planting pattern but not by fertilization (Supplementary Table S4). Intercropping significantly decreased the gene copies of *F. oxysporum* over monoculture by 2.91, 7.33 and 9.56% in bulk soil, rhizosphere soil and root endosphere, respectively (Figure 2C). The suppression of *F. oxysporum* was much stronger in faba bean than in maize, especially in the RE (Supplementary Figure S1). Further analysis showed negative correlations between the gene copies of *F. oxysporum* and faba bean yields in the P fertilization treatment but not in the zero P treatment (Figure 2D). No correlation was observed in maize plants.

### Bacterial diversity and community composition

**Figure 3:**
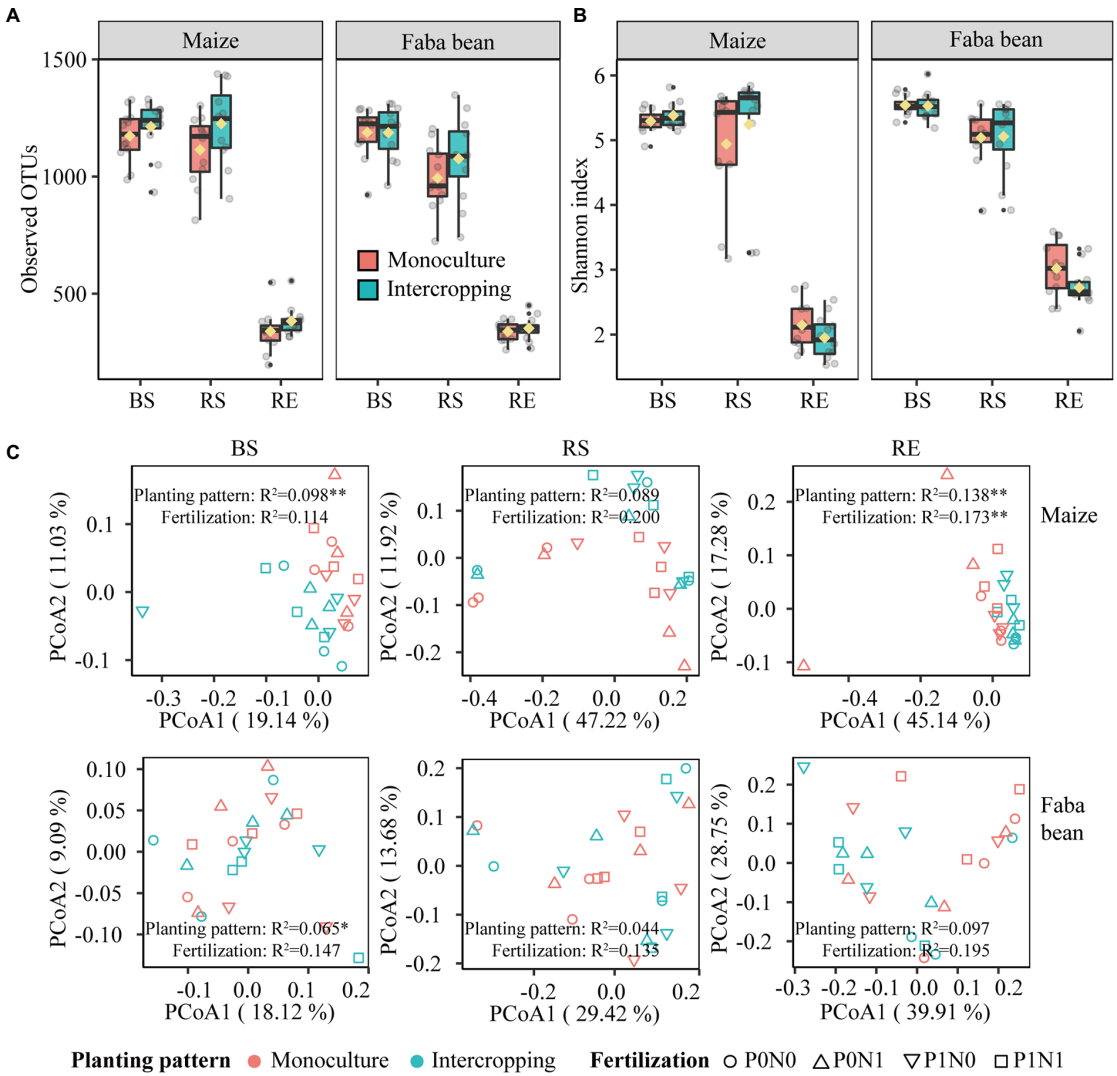
Bacterial alpha diversity of observed OTUs **(A)** and Shannon index **(B)** in bulk soil (BS), rhizosphere soil (RS) and root endosphere (RE) of maize and faba bean as affected by planting pattern. Bacterial community composition in bulk soil, rhizosphere and root endosphere as affected by planting pattern and fertilization **(C)**. P0N0, no fertilization; P0N1, sole N fertilization at 180 kg N ha^−1^ yr.^−1^ as urea; P1N0, sole P fertilization at 40 kg P ha^−1^ yr.^−1^ as superphosphate; P1N1, N and P fertilization at 180 kg N ha^−1^ yr.^−1^ and 40 kg P ha^−1^ yr.^−1^ respectively; ^*^p < 0.05; ^**^p < 0.01.

Irrespective of fertilization, intercropping increased bacterial OTUs observed and Shannon index in both plant species at marginal levels, with more pronounced effects on the maize rhizosphere (Figures 3A,B). Planting pattern had marginal effects on the observed OTUs and Shannon index in the rhizosphere soils of maize and faba bean (*p* < 0.1), and only the Shannon index in maize RE was significantly affected by fertilization (*p* < 0.05; Supplementary Table S5). Based on the PCoA profiles, bacterial communities were clearly differentiated, separated by compartment and crop species. This was further supported by the PERMANOVA analysis based on a Bray–Curtis distance metric (compartment: R^2^ = 0.514, *p* < 0.01; crop species: R^2^ = 0.092, *p* < 0.01, Supplementary Figure S2 A). PCoA analysis shows that the bacterial communities in BS, RS and RE of maize and faba bean were significantly affected by planting pattern, and the effect of fertilization was not significant except in maize RE (Figure 3C). In maize BS and RE, bacterial profiles in monoculture were clustered together and separated from intercropping along PCoA 1 (*p* < 0.01). In faba bean, bulk soil samples were significantly affected by planting pattern (*p* < 0.05) but not by fertilization. Maize RS and faba bean RE were marginally significantly affected by planting pattern (maize, *p* = 0.051; faba bean, *p* = 0.051) and fertilization treatment (maize, *p* = 0.074; faba bean, *p* = 0.083). Similarly, at family level the abundant taxa present in all samples were more significantly affected by planting pattern than by fertilization (Supplementary Figure S2 B; Supplementary Table S6). In addition, intercropping increased bacterial network complexity, as shown by the higher average degree, network density and more hub nodes compared to the corresponding monocultures (Supplementary Figure S3; Supplementary Tables S7, S8). Details of bacterial diversity, community structure and bacterial network are shown in the Supplementary Results.

### Siderophore-producing rhizobacteria and microbial functional genes in the rhizosphere of both plant species

**Figure 4:**
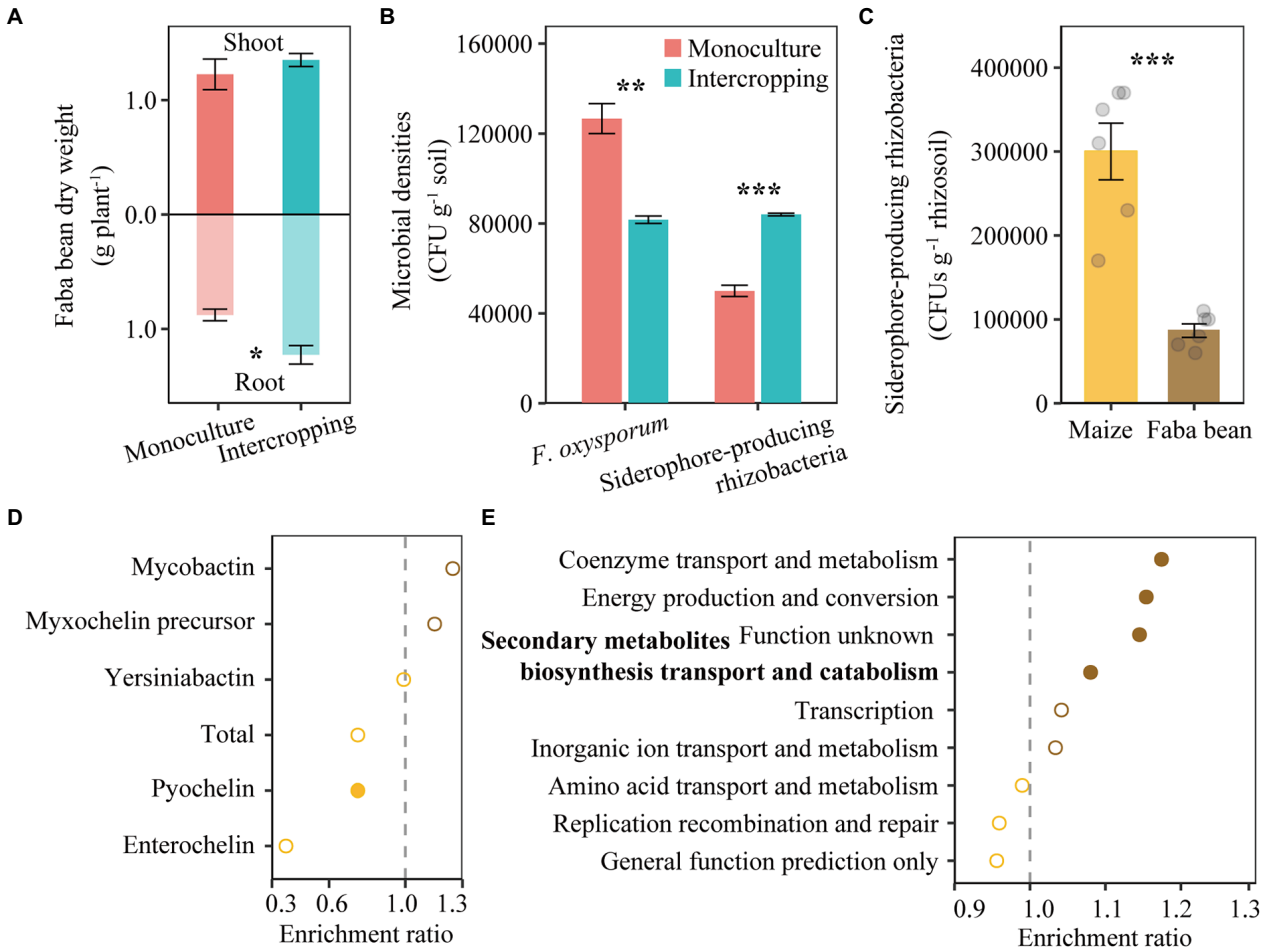
Shoot and root dry weights of faba bean in monoculture and intercropping **(A)**. Abundance of cultivable *F. oxysporum* and siderophore-producing rhizobacteria in faba bean rhizosphere in the pot experiment **(B)**. Number of siderophore-producing rhizobacteria in rhizosphere of maize and faba bean in the field experiment **(C)**. Relative abundance of nonribosomal peptide synthetase (NRPS) gene in maize and faba bean rhizosphere soil based on metagenome sequencing **(D)**. Bacterial gene ontology (GO) analysis for all genes related to iron metabolism, showing enriched (right next to the dash line) or depleted (left next to the dash line) in faba bean rhizosphere **(E)**. In panel **(A,B)**, data (*n* = 3; mean ± S.E.) of faba bean samples collected from monoculture and intercropping are presented. In panel **(C–E)**, data (*n* = 6; mean ± S.E.) of rhizosphere soil samples collected from maize and faba bean rhizosphere are presented. Student’s *t*-test was conducted to check the differences between two group samples. ^*^p < 0.05; ^**^p < 0.01; ^***^p < 0.001.

A pot experiment was conducted to examine the effect of intercropping on the reduction of pathogens. Intercropping increased dry weights of faba bean, especially root dry weights (Figure 4A). The abundance of *F. oxysporum* decreased but the numbers of siderophore-producing rhizobacteria increased in intercropping than in monoculture of faba bean (Figure 4B). The number of siderophore-producing rhizobacteria increased by 71.1% in RS of faba bean compared to that of maize (Figure 4C).

In the field experiment, metagenomic analysis of rhizosphere soil in maize and faba bean showed that the total gene abundance of pyochelin biosynthesis was enriched in the RS of maize compared to that of faba bean (Figure 4D), in particular the *pchF* gene. In addition, the abundance of NRPS genes involved in enterochelin biosynthesis (e.g., *entD, entF* and *entA*) and mycobactin biosynthesis (*mbtI*) were significantly higher in maize RS, while *mbtG* involved in mycobactin biosynthesis was higher in faba bean RS. The gene *irp5* involved in yersiniabactin biosynthesis was found only in faba bean RS (Supplementary Figure S4 A).

As we targeted iron-related microbial gene functions, we sorted the COGs likely related to iron metabolism. Coenzyme transport and metabolism, energy production and conversion, and secondary metabolite biosynthesis, transport and catabolism were significantly enriched in faba bean RS (Figure 4E). Of all COGs likely related to iron metabolism, 12 were enriched in faba bean RS but only three were enriched in maize RS (Supplementary Figure S4 B). The COGs involved in coenzyme transport and metabolism (e.g., COG1072 and COG2138), energy production and conversion (e.g., COG1333, COG1251 and COG1018, 0.0676% in faba bean RS and 0.0575% in maize RS), secondary metabolite biosynthesis transport and catabolism (COG2124, 0.126% in faba bean RS and 0.116% in maize RS), inorganic ion transport and metabolism [e.g., nitrate/nitrite transporter NarK (COG2223) and catalase (peroxidase I, COG0376)] and unknown functions (COG3544) were enriched in faba bean RS. By contrast, amino acid transport and metabolism and replication recombination and repair were slightly enriched in maize RS. Other COG0065 (homoaconitase/3-isopropylmalate dehydratase large subunit) for amino acid transport and metabolism, COG1199 (rad3-related DNA helicase) for replication recombination and repair and COG1032 (radical SAM superfamily enzyme YgiQ, UPF0313 family, 0.077% in maize RS and 0.068% in faba bean RS) for general function prediction were enriched only in maize RS.

### Isolate screening and identification of antagonistic strains

**Figure 5:**
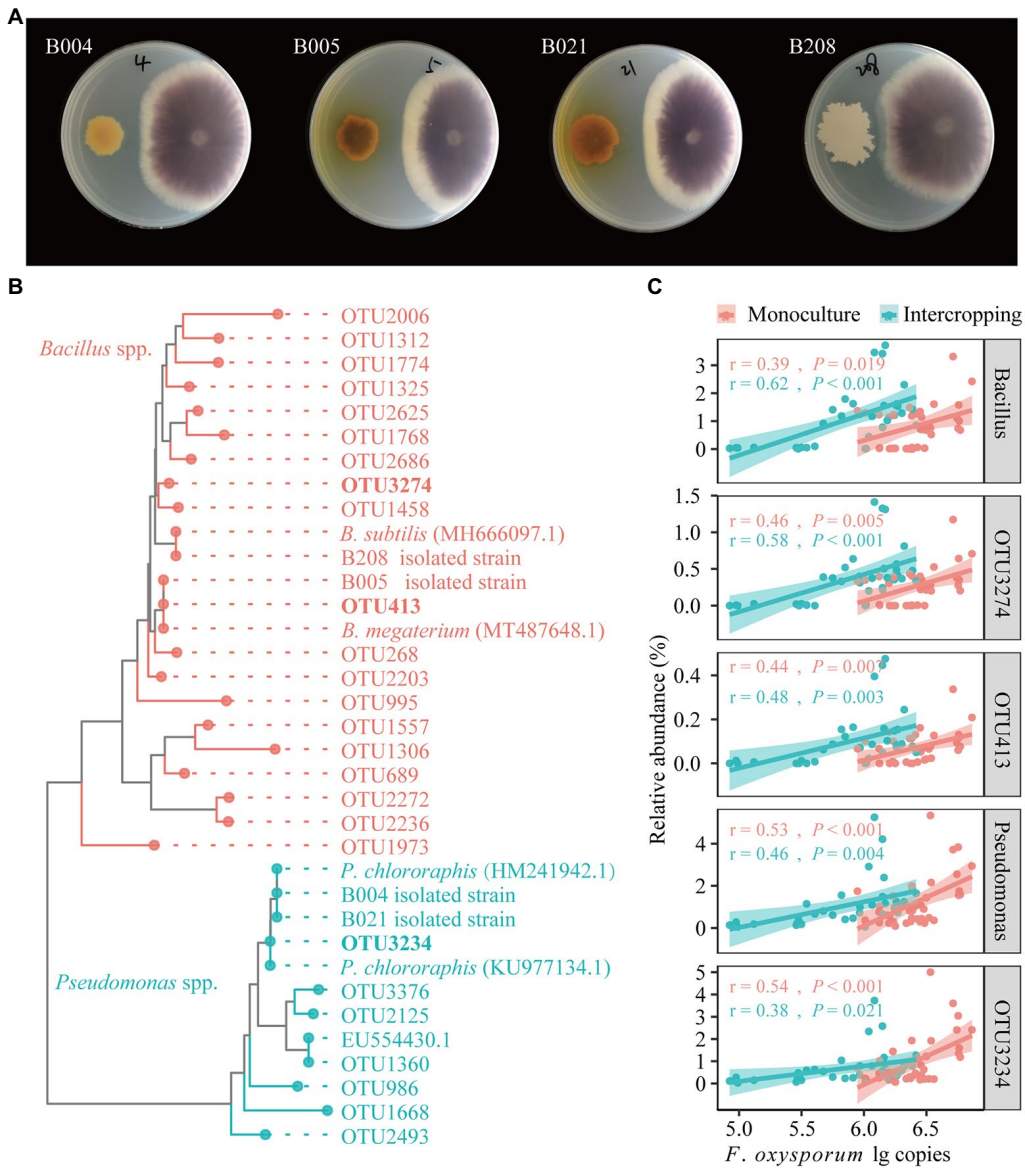
Dual culture assays for *in vitro* inhibition of FOF growth by bacterial isolates **(A)**. Neighbor-joining (NJ) phylogenetic tree shows the four isolates B004, B021, B005 and B208 with representative sequences selected from GenBank (bold text) and representative sequences of OTUs affiliated with *Bacillus* and *Pseudomonas* **(B)**. Correlations between the relative abundances of the antagonistic OTUs, *Bacillus* and *Pseudomonas* with the gene copies of *F. oxysporum* across all samples **(C)**.

A total of 95 bacterial strains were isolated from bulk soils in maize/faba bean intercropping system in the field. The isolates were grouped into 13 genera, mainly *Pseudomonas* (36), *Escherichia* (20), and *Bacillus* (13), and the less abundant groups of *Klebsiella*, *Acinetobacter*, *Pantoea*, *Chitinophaga*, *Lysobacter*, and *Stenotrophomonas* (Supplementary Table S9). In the dual culture experiments, four isolates (B004, B005, B021, and B208) showed strong antagonistic effects on FOF growth based on visual agar observation (Figure 5A). The constructed phylogenetic tree indicates that the four antagonistic isolates showed high similarity to the reference strains for *Pseudomonas* (B004 and B021) and *Bacillus* (B005 and B208), respectively (Figure 5B). *B. megaterium* (B005) was grouped together with OTU413, *B. subtilis* (B208) with OTU3274, and *P. chlororaphis* (B004 and B021) with OTU3234. Importantly, OTU413 was the hub node in the bacterial network. Correlation analysis showed that the relative abundances of *Bacillus* (OTU413, OTU3274) and *Pseudomonas* (OTU3234) were positively correlated with *F. oxysporum* gene copies in the BS, RS and RE of faba bean irrespective of planting pattern (Figure 5C).

### *In vitro* antagonistic activities of isolates against FOF and the inoculation experiment

**Figure 6:**
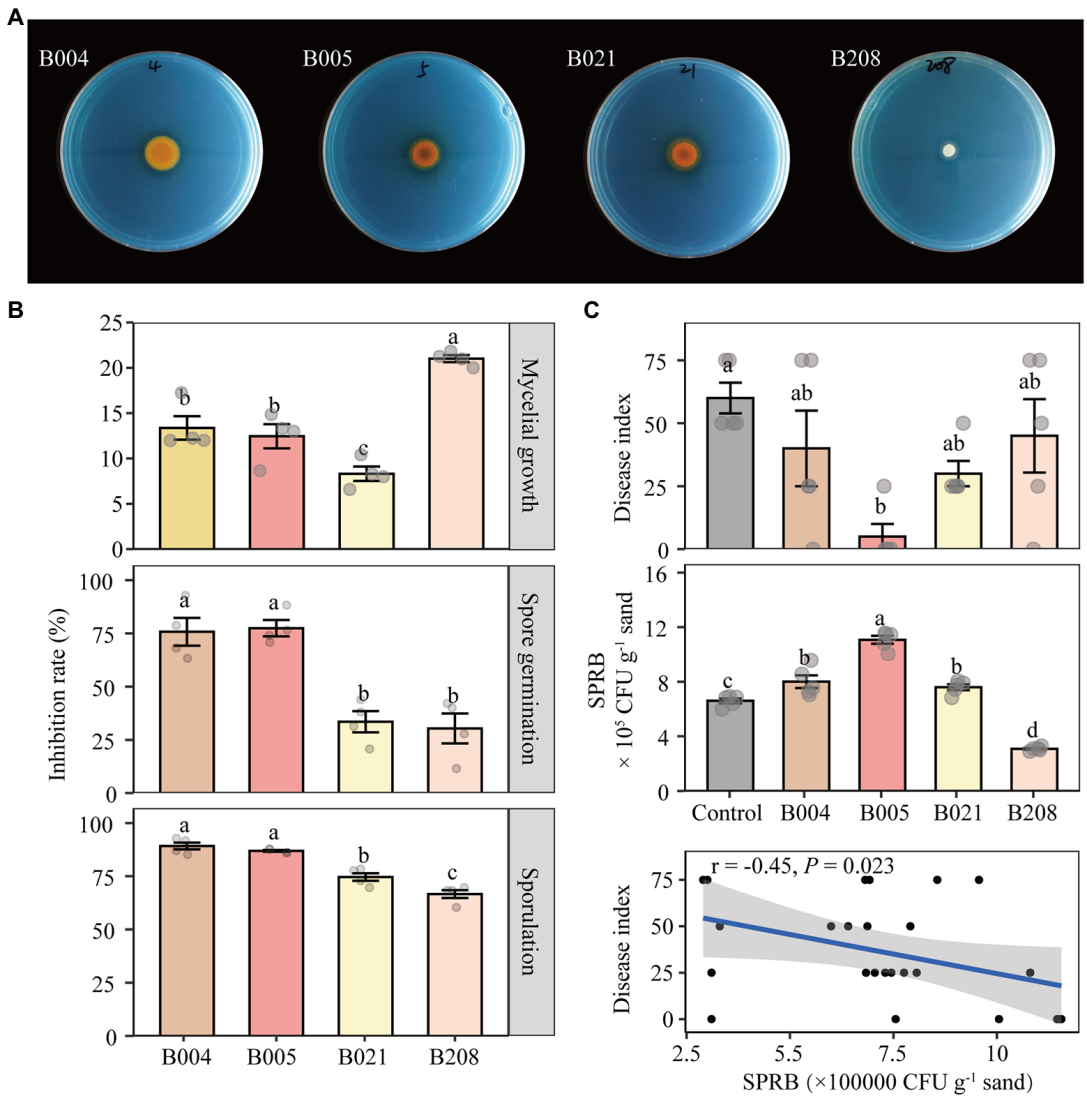
Siderophore-producing capability of the four antagonistic isolates **(A)**. Effect of metabolites of the four isolates on mycelial growth, spore germination and sporulation of FOF **(B)**. Disease index of Fusarium wilt and the number of siderophore-producing rhizobacteria (SPRB) in the control (sterilized water) and inoculated treatments B004, B005, B021 and B208, and the correlation of the two indicators **(C)**. B004 and B021 are affiliated with *Pseudomonas chlororaphis*; B005 with *Bacillus megaterium* and B208 with *Bacillus subtilis*. Different lowercase letters in each plot indicate significant differences according to Duncan’s multiple range test at *p* < 0.05.

*In vitro* analysis was conducted to quantify the inhibitory effect of the four antagonistic bacteria on FOF. The inhibition rates of B004, B005, B021, and B208 on FOF mycelial growth were 45.2, 55.2, 54.2, and 33.8%, respectively (Supplementary Figure S5 A). In addition, B005 and B021 were effective in siderophore production as shown by the orange hole on the chrome azurol S medium (Figure 6A). In terms of FOF spore germination and sporulation, all four isolates showed strong inhibitory effects (Figure 6B). Compared to B021 and B208, the effects of B004 and B005 on suppressing mycelial growth of *F. oxysporum* were stronger but their metabolites were weaker.

The disease suppressive activity of the four isolates against FOF was further investigated in a bioassay experiment. Compared to the control, the disease index of faba bean plants inoculated with B005 and B021 decreased by 75 and 50%, whereas those inoculated with B004 and B208 decreased by 33 and 25%, respectively (Figure 6C). In addition, the CFU number of siderophore-producing rhizobacteria in the B005 inoculation treatment was the highest, followed by B004 and B021, while the number in the B208 treatment was the lowest. The disease index of Fusarium wilt was significantly negatively correlated with the CFU numbers of the siderophore-producing rhizobacteria (*r* = −0.45, *p* = 0.023). However, no significant differences in the biomass of faba bean were detected (Supplementary Figure S5 B).

## Discussion

In this study, overyielding was in line with a recent meta-analysis showing that maize was the major contributor to overyielding in most intercropping studies (Li et al., 2020). Facilitation is well demonstrated in cereal/legume systems for mutual benefit in terms of N and P nutrient use (Li et al., 2007, 2016). In addition to nutrient supply mechanisms, decreasing the abundance of potential pathogens such as *F. oxysporum* in intercropping may also attribute to yield advantage (Figure 2), though it is difficult to directly quantify the contribution of disease suppression to yield increase under field conditions. On the one hand, there may be a “dilution” effect because intercropping increased bacterial diversity (OTU richness and Shannon index) in nearly all compartments of maize and faba bean (Figures 3A,B), consisting with enhanced microbial biodiversity and grain yields in other intercropping studies (Zhang et al., 2020; Li et al., 2021b). On the other hand, the decline in pathogen abundance may be the result of increased network complexity and enhanced competitiveness against pathogens in intercropping (Supplementary Figure S3). Here, we found that bacterial networks in intercropping had higher complexity and higher connectivity than those in monoculture, indicating the potential to suppress fungal pathogens of focal plants by introducing microbiome of the non-host plants. Similar results were obtained in pea/wheat intercropping (Pivato et al., 2021), and also in monocultures of olive cultivars exposed to verticillium wilt (Fernandez-Gonzalez et al., 2020) and in tobacco plants to wilt disease (Yang et al., 2017). Together, diversified cropping system such as intercropping, rotation (Zhou et al., 2017), and companion planting (Fu et al., 2015) is an effective approach to reduce pathogen abundance and disease incidence.

Soil nutrient supply and especially iron supply affects the pathogenicity of plant pathogens (Gu et al., 2020). Iron availability is particularly low on the calcareous soil, though the crops did not exhibit visible iron deficiency symptoms. In our second hypothesis, we speculated that the rhizosphere microbiome from neighboring maize plants might have direct effects on the disease suppression of faba bean plants, likely due to differences in the abundance of iron siderophore-producing bacteria. Our results partly support this hypothesis. First, the abundance of the siderophore-producing rhizobacteria was significantly higher in maize rhizosphere than in faba bean rhizosphere (Figure 4C), and it was higher in intercropping than in monocultured faba bean RS (Figure 4B). Second, results from the *in vivo* experiment showed that the two isolates B005 and B021 had higher siderosphore-producing capacity and showed the highest suppression of FOF among the four selected isolates in terms of disease index (Figure 6). Finally, results from metagenomics demonstrated that the nonribosomal peptide synthetases genes, in particular pyochelin (e.g., *pchF*), were significantly higher in the maize rhizosphere relative than in the faba bean rhizosphere. The genes for enterochelin biosynthesis (e.g., *entF*, *entD* and *entA*) and total pyochelin biosynthesis showed similar trends though not statistically significant (Figure 4; Supplementary Figure S4). This may be associated with the rhizosphere traits of the two crops. Maize is a strategy II species and faba bean is strategy I (Römheld and Marschner, 1986). A significant decrease in soil pH and increase in organic acids with faba bean roots may be the major determinants modulating the assembly and functions of bacteria in the rhizosphere (Liao et al., 2022; Meng et al., 2022). Further studies are needed to unravel the microbial interactions mediated by nutrient cues of different crop species.

The bacterial community composition was altered in intercropping compared to monocultures of both maize and faba bean. This agrees with previous studies in which intercropping altered rhizosphere microbial communities of proso millet, mung bean (Dang et al., 2020), and wheat and faba bean (Granzow et al., 2017). Similar to previous studies (Zheng and Gong, 2019; Xiong et al., 2021), compartment and host identity but not fertilization were the predominant factors attributable to distinct changes in bacterial community composition (Supplementary Figure S2 A; Figure 3; Supplementary Table S6). In particular, *Bacillaceae* was more abundant in RS of maize than in that of faba bean, but *Pseudomonadaceae* and *Rhizobiaceae* showed the opposite trend (Supplementary Figure S2 B). *Rhizobiaceae*, *Bacillaceae* and *Pseudomonadaceae* are regarded as plant growth promoting bacteria (Zarraonaindia et al., 2015), and plants are shown to recruit specific taxa to suppress disease (Mendes et al., 2011; Carrión et al., 2019). We identified 60 hub nodes belonging to 52 OTUs as keystone taxa in the bacterial network and these taxa belonged to *Bacillaceae*, *Sphingomonadaceae*, *Lysobacter*, *Streptomycetaceae*, *Xanthomonadaceae* and others (Supplementary Table S8). Some of these taxa are widely recognized to play an important role in regulating plant fitness, inhibition of pathogens and increased tolerance (Mendes et al., 2011; Gomez Exposito et al., 2017). After testing 95 viable colonies, we found that two *Bacillus* isolates (B005 and B208) and two *Pseudomonas* isolates (B004 and B021) showed strong antagonistic effects on FOF (Figure 5A). *Bacillus* spp. and *Pseudomonas* spp. are known to be biocontrol agents, showing strong antagonistic effects on plant pathogens including FOF (Berendsen et al., 2012; Tao et al., 2020). The greenhouse bioassay showed that B005 and B021 significantly reduced the disease index by 75 and 50%, respectively, while the other two isolates did not have such efficacy (Figure 6C). As the experimental period was relatively short, no significant difference was observed in biomass among different treatments, despite of the inoculation with antagonistic bacteria showing a tendency to promote biomass (Supplementary Figure S5 B). Interestingly, we found that isolate B005 (OTU413) appeared to be highly affiliated with keystone taxa in the empirical bacterial network (Supplementary Figure S3; Figure 5). The results indicate that keystone taxa in the bacterial network are likely to be important in determining species interactions and microbe-pathogen interactions. As individual isolate showed different effects on the disease index of faba bean (Figure 6C), these results indicate that a synthetic community may be more promising in the manipulation of pathogen control, although single isolate or simplified synthetic community inoculation has also been reported (Yin et al., 2021; Li et al., 2021c).

## Conclusion

**Figure 7:**
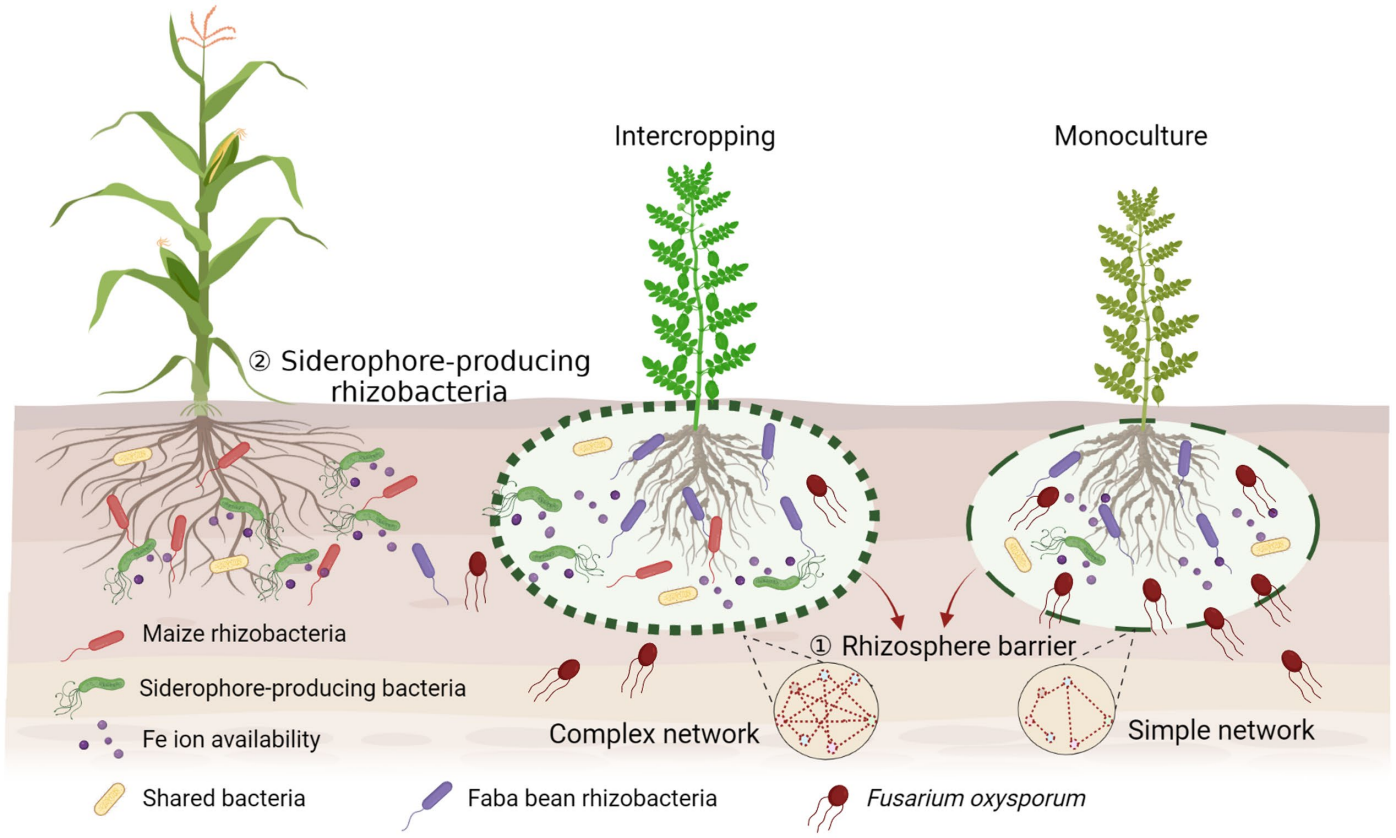
Conceptual model displaying the potential mechanisms of suppression of the fungal pathogen in the intercropping rhizosphere by building up a complex bacterial network to form the rhizosphere barrier and simulating more siderophore-producing bacteria to compete with pathogenic fungi for iron. Created with BioRender.com.

Increasing evidence emphasizes the importance of soil microbiota in the relationships of plant diversity-ecosystem productivity in intercropping systems. Microbial interactions in intercropping are superimposed and are more complex as they involve multiple players. Our results indicate a new perspective in rhizosphere microbial ecology by providing evidence that interactions between rhizosphere beneficial microbes and pathogens contribute to the suppression of plant pathogens by building up a complex bacterial network to form a rhizosphere barrier, and stimulating siderophore-producing bacteria to compete with pathogenic fungi for iron, then increasing the production of the host plants (Figure 7). This mechanism can be extrapolated to the development of proper intercropping systems. Our study provides evidence for direct facilitative interactions between beneficial microbes of one crop to pathogen suppressiveness of the neighboring crop in explaining overyielding advantages in intercropping. This has broad agronomic implication to enhance ecosystem services in intercropping through the manipulation of microbial interactions in addition to facilitative root–root interactions.

## Data availability statement

The data presented in the study are deposited in the Genome Sequence Archive repository, accession number CRA004524 and CRA005329.

## Author contributions

XS conducted the experiments and analyzed the data. CZ coordinated the long-term field experiments. SB analyzed the metagenome data. JZ conceived the study and supervised the project. GW helped with the data analysis. All authors contributed to the article and approved the submitted version.

## Funding

This work was funded by the National Key Research and Development Program of China (2017YFD0202102) and the National Natural Science Foundation of China (31872182).

## Conflict of interest

The authors declare that the research was conducted in the absence of any commercial or financial relationships that could be construed as a potential conflict of interest.

## Publisher’s note

All claims expressed in this article are solely those of the authors and do not necessarily represent those of their affiliated organizations, or those of the publisher, the editors and the reviewers. Any product that may beevaluated in this article, or claim that may be made by its manufacturer, is not guaranteed or endorsed by the publisher.

## Footnotes

^1^ http://www.r-project.org/
