# Supplementary material for: High bacterial diversity and siderophore-producing bacteria collectively suppress Fusarium oxysporum in maize/faba bean intercropping
Source: Front Microbiol. 2022 Aug 5;13:972587. doi: 10.3389/fmicb.2022.972587 (PMC9389221; doi:10.3389/fmicb.2022.972587)
Supplement: Supplementary file 1 [file Data_Sheet_1.docx]

Supplementary Material

High bacterial diversity and siderophore producing bacteria collectively suppress *Fusarium oxysporum* in maize/faba bean intercropping

Xinzhan Sun^1†^, Chaochun Zhang^1†^, Shuikuan Bei^1^, Guangzhou Wang^1^, Stefan Geisen^2^, Laurent Bedoussac^3^, Peter Christie^1^ and Junling Zhang^1*^

^1^College of Resources and Environmental Sciences; National Academy of Agriculture Green Development; Key Laboratory of Plant-Soil Interactions, Ministry of Education, China Agricultural University, Beijing 100193, China

^2^Laboratory of Nematology, Wageningen University, Wageningen 6700 AA, The Netherlands

^3^AGIR, University of Toulouse, ENSFEA, INRAE, Castanet-Tolosan, France

*** Correspondence:**Junling Zhang
Junlingz@cau.edu.cn

**Supplementary materials and methods**

Field management

The management of the field experiment was described in Liao et al. (2021). Briefly, each field plot was 4.2 m wide and 8.0 m long. Maize was planted with 0.4 m of row distance and 0.3 m of plant spacing, and the density was 8.33 plants m^-2^. Faba bean was planted with 0.2 m of both row distance and plant spacing, and the density was 25 plants m^-2^. The densities of both maize and faba bean remained constant between monoculture and intercropping. Each intercropped plot consisted of three strips and each strip was 1.4 m wide containing two rows of maize and three rows of faba bean. Nitrogen (N) was applied as urea at 180 kg N ha^-1^ yr^-1^, with half the total N broadcast as basal fertilizer before sowing of the seeds and the remainder side-dressed at the V12 stage of maize plants. Phosphorus (P) was applied as superphosphate at 40 kg P ha^-1^ yr^-1^, and potassium (K) was applied as potassium sulfate at 50 kg K ha^-1^ yr^-1^, and both were broadcasted as basal fertilizers. No N or P fertilizers were applied to control plots of zero fertilization. Only N or P fertilizers were supplied to the sole N or P fertilization treatments. Both N and P fertilizers were supplied in the NP fertilization treatment. All plots were irrigated during the growing season to prevent water stress and the weeds were manually removed from the fields. After harvest, the straw was completely removed while root residues were remained in the field. The plots remained fallow from October until March of the following year.

**Field sampling**

Samples were collected from monocultured and intercropped maize and faba bean in June 2019, at the V10 growth stage of maize and the flowering stage of faba bean. Maize and faba bean were sampled from both monoculture and intercropping plots. In each plot, three plants were randomly selected, and the shoots were cut 2 cm above the ground surface. The roots were carefully excavated with a shovel and shaken by hand. The soil that easily detached from the roots was defined as bulk soil (BS) and the soil adhering to the roots was defined as rhizosphere soil (RS). Rhizosphere soil was washed off the roots by vigorous shaking in single strength phosphate buffer solution (PBS) followed by centrifugation at 4000 rpm for 5 min. Then the roots were successively washed with 70% (v/v) ethanol and 30% (v/v) H_2_O_2_, and then rinsed repeatedly with sterilized water to remove all root surface-attached DNA. The cleaned root samples were stored at -80 °C until DNA extraction and regarded as root endosphere (RE). Both bulk and rhizosphere soils were vacuum-dried at -20 °C then stored at -80 °C until DNA extraction. The remainder were stored at 4 °C and air-dried at room temperature for determination of physicochemical properties.

**Determination of soil physicochemical properties**

Selected physicochemical characteristics in the bulk soil were determined. Briefly, soil pH was determined in a soil suspension with a soil:water ratio of 1:2.5 (w/v) using a pH electrode (SevenCompact pH meter S220; Mettler Toledo, Columbus, OH). Soil total C (TC) and total N (TN) were determined by Elemental Analyser (EA1108; Carlo Erba, Turin, Italy) using the Dumas combustion method. Dissolved organic carbon (DOC) was extracted with 0.5 mol L^-1^ K_2_SO_4_, passed through a 0.45-μm pore membrane filter and determined using a liquid analyzer (TOC-VCPH; Shimadzu, Kyoto, Japan) (Jones and Willett 2006). Fresh soils were extracted for the analysis of ammonium-N (NH_4_^+^-N) and nitrate-N (NO_3_^-^-N) contents using 0.01 mol L^-1^ CaCl_2_ solution at a ratio of 12:100 (w/v). Nutrient contents were determined using continuous flow analysis (TRAACS 2000, Bran and Luebbe, Norderstedt, Germany). Available P (AP) was determined by the colorimetric method, with 2.5 g air dried soil extracted in 0.5 mol L^-1^ NaHCO_3_ solution (Olsen and Sommers 1982). Soil moisture content was gravimetrically measured by oven-drying the fresh soil samples at 105 °C to constant weight.

**DNA extraction and real-time quantitative PCR**

DNA extraction was conducted using a fast DNA SPIN Kit (MP Biomedicals, Cleveland, OH). 0.5 g dried soil from the fields was used for BS and RS, and 0.2 g fresh root samples for RE. The quantity and concentration of DNA samples were determined using a Nanodrop ONE spectrophotometer (Nanodrop Technologies, Wilmington, DE). Real-time quantitative PCR (qPCR) was conducted to measure the gene copies of *Fusarium* *oxysporum* using primer pairs ITS1F (5’-CTTGGTCATTTAGAGGAAGTAA-3’) / AFP308R (5’-CGAATTAACGCGAGTCCCAAC-3’) on a CFX-96 thermocycler (Bio-Rad Laboratories, Hercules, CA) (Gardes and Bruns 1993; Lievens et al., 2005). The reaction mixture (20 μL) comprised 10 μL of Premix Ex Taq™ (2×) (Takara Bio, Kyoto, Japan), 0.5 μL of each of the two primers (10 μM), and 1 μL of DNA extract under the following thermal conditions: 2 min at 95 °C, 40 cycles of 15 s at 94 °C, 15 s at 58 °C, and 10 s at 72 °C. The amplification efficiency was > 85% and the specificity of qPCR reactions was assessed by melting curve analysis from 65 to 95 °C. Standard curves were generated with a 10-fold dilution series of plasmid DNA containing fragments of the ITS region of *F.* *oxysporum* from soil samples. Finally, the *F.* *oxysporum* lg copies were calculated according to the standard curve.

**Bacterial PCR amplification and high-throughput sequencing of the 16S rRNA gene**

The interference by plant chloroplasts was eliminated by first using 799F (5´-AACMGGATTAGATACCCKG-3´) and 1392R (5´-ACGGGCGGTGTGTRC-3´) to amplify the bacterial 16S rRNA gene (Beckers et al., 2017). Briefly, 50 μL PCR reaction contained 44 μL of 1× TaqMix (TsingKe Biotechnology, Beijing, China), 2 μL of each primer (10 μM), and 2 μL of template DNA. The PCR was conducted following the program: 3 min of denaturation at 95 °C, 27 cycles of 95 °C for 30 s, 55 °C for 30 s, and 72 °C for 45 s, and a final extension at 72 °C for 10 min. Products of the first PCR were used as a template for the 2nd PCR amplification with the primers 799F (5´-AACMGGATTAGATACCCKG-3´) and 1193R (5´-ACGTCATCCCCACCTTCC-3´) targeting the V5-V7 region (Zhang et al., 2019). The second PCR reactions followed the same program as the first PCR except only 13 cycles were conducted. Finally, PCR products were purified and mixed in equimolar ratios to obtain a quantitative sample DNA library which was further used for sequencing from the adaptor. The purified PCR products were sequenced using the Illumina MiSeq PE300 platform (Illumina, San Diego, CA).

**Bioinformatics analysis**

Raw sequence data were deposited in the Genome Sequence Archive (GSA, Genomics, Proteomics & Bioinformatics 2017) under the accession number CRA004524 accessible at <https://ngdc.cncb.ac.cn/gsa>. Raw sequences of the bacterial 16S rRNA gene were quality-filtered using Trimmomatic version 0.33 with default parameters (Bolger et al., 2014). Afterwards the quality-filtered paired-end sequences were merged using FLASH version 1.2.9 with default settings (Magoc and Salzberg 2011). The operational taxonomic units (OTUs) were clustered with 97% similarity cutoff using UPARSE series of scripts with a novel ‘greedy’ algorithm that conducts chimera filtering and OTU clustering simultaneously (Edgar 2013). Each 16S rRNA gene sequence was taxonomically assigned using the RDP Classifier version 2.2 against the SILVA database (SSU138) with a confidence threshold of 70% (Wang et al., 2007). All non-bacterial taxa were removed resulting in 3,751,919 (377 bp average length) high-quality sequences that were clustered into 3390 OTUs.

**Co-occurrence network analysis**

In the field experiment, the microbial interactions between monoculuture and intercropped maize and faba bean were compared by depicting co-occurrence network analysis among bacterial taxa through network analysis using the “WGCNA” package based on Spearman’s correlation matrices (Langfelder and Horvath 2012). Only OTUs with relative abundance > 0.1% were used for microbial community analysis to reduce the complexity of calculation and to ensure the accuracy of results (Klaedtke et al., 2016). A co-occurrence was considered to be robust if the Spearman’s correlation coefficient was > 0.60 and *P* < 0.01. The *P* values were adjusted using the Benjamini–Hochberg procedure to minimize false-positive signals (Benjamini and Hochberg 1995). The *cluster_fast_greedy* function in the “igraph” package was used to calculate network modules (Hassani et al., 2018). The networks were visualized using the interactive platform Gephi (Bastian et al., 2009). In addition, random networks with the same numbers of nodes and edges as the empirical network were generated, and topological indices were summarized by 999 iterations based on the Erdös-Réyni model to determine if the network properties were prone to errors (Erdos 1970).

The network properties were obtained using the “igraph” package (Csardi and Nepusz 2006). The calculated topological characteristics of bacterial networks comprised average degree, network density, clustering coefficient and positive proportion. Average degree refers to the average connection of each node with another unique node in the network; network density refers to the intensity of connections among nodes; and clustering coefficient represents the degree to which the nodes tend to cluster together (Jiao et al., 2020). Therefore, with greater average degree, network density and clustering coefficient together indicate a higher connected network (Zhao et al., 2019). In addition, lower average path lengths and network diameters indicated closer associations in the network (Ma et al., 2016). Here, OTUs with degree > 85 were selected as hub nodes representing potential keystone taxa (Hartman et al., 2018).

**Supplementary results**

Physicochemical properties of the bulk soil

Soil pH was consistently lower in the intercropping (pH 7.49) than in the monoculture (pH 7.62) regardless of fertilization treatment, while AP was the reverse (**Table S3**). The NH_4_^+^-N concentration was decreased while NO_3_^-^-N was increased in the intercropping of faba bean than in the monoculture except in the NP fertilization treatment. TC and TN contents were variable and showed no consistent pattern. The tested parameters in maize plants were generally higher in the intercropping than in the monocropping treatment, with no signficant differences among fertilization treatments except for AP concentration. Phosphorus fertilization increased the AP concentration in both monoculture and intercropping treatments, by 8.31 and 5.80 times in maize and by 8.76 and 6.47 times in faba bean.

Bacterial community structure

Bacterial communities were clearly differentiated based on the PCoA profiles, separated by compartment (R^2^ = 0.514) and crop species (R^2^ = 0.092), followed by fertilization (R^2^ = 0.021) and planting pattern (R^2^ = 0.010) (*P* < 0.01, Figure S2a). The OTUs were clustered into 236 families, of which the 21 most abundant families were shown in Figure S2b. The most abundant family was *Enterobacteriaceae* (13%), followed by *Rhizobiaceae*, *Comamonadaceae*, *Sphingomonadaceae* and *Streptomycetaceae* (5.22, 3.78, 3.36 and 2.74%, respectively). Significant changes due to intercropping were observed in *Rhizobiaceae* and *Streptomycetaceae. Rhizobiaceae* was enriched in RE of intercropped faba bean and *Streptomycetaceae* was enriched in RS of maize and RE of faba bean in monoculture. Signficant differences between maize and faba bean were observed in the RS compartment. *Bacillaceae* was enriched in maize and *Flavobacteriaceae* was in RS of faba bean. At the family level, bacterial communities in each compartment of maize and faba bean were significantly affected by planting pattern, followed by fertilization (Table S6). In intercropped maize, *Flavobacteriaceae* was enriched in BS, *Gemmatimonadaceae* in RS, *Enterobacteriaceae* and *Streptomycetaceae* in RE, and *Pseudomonadaceae* in both BS and RS. In intercropped faba bean, *Geminicoccaceae* was enriched in RE and *Pseudomonadaceae* in BS. By contrast, in intercropped maize plants, *Rhizobiaceae* was declined in RS, *Sphingomonadaceae* and *Streptomycetaceae* in BS and RS, *Nocardioidaceae* in BS, and *Pseudonocardiaceae* in all three compartments. In intercropped faba bean, *Streptomycetaceae* was declined in all three compartments, *Nocardioidaceae* in RE, and *Micromonosporaceae* and *Oxalobacteraceae* in BS.

**Bacterial network in monoculture and intercropping**

As the fertilization effect was relatively weak, we constructed the co-occurrence networks of bacteria in monoculture and intercropping of maize and faba bean with pooled data across different compartments and fertilization treatments. The structural properties of the real-world bacterial co-occurrence networks were greater (clustering coefficient > 0.7, modularity > 0.1) than those of an identically sized random Erdös–Réyni network (**Table S7**). In both maize and faba bean, compared to the corresponding monoculture, intercropping increased network complexity, as shown by the higher average degree (maize, 63.7 vs 53.5; faba bean, 56.0 vs 51.9), network density (maize, 0.47 vs 0.41; faba bean, 0.40 vs 0.37) and by the slight increase in clustering coefficient in intercropping (**Figure S3**). The numbers of hub nodes in intercropping were higher than in monocultures of maize (44 vs 1) or faba bean (13 vs 2), of which *Bacillus* (OTU1774, OTU2203, OTU1458, OTU413), *Sphingomonas* (OTU12, OTU1944, OTU1942), *Lysobacter* (OTU3062) and *Streptomyces* (OTU1720) were more abundant in intercropping than in monoculture (**Table S8**).

**Supplementary figures**


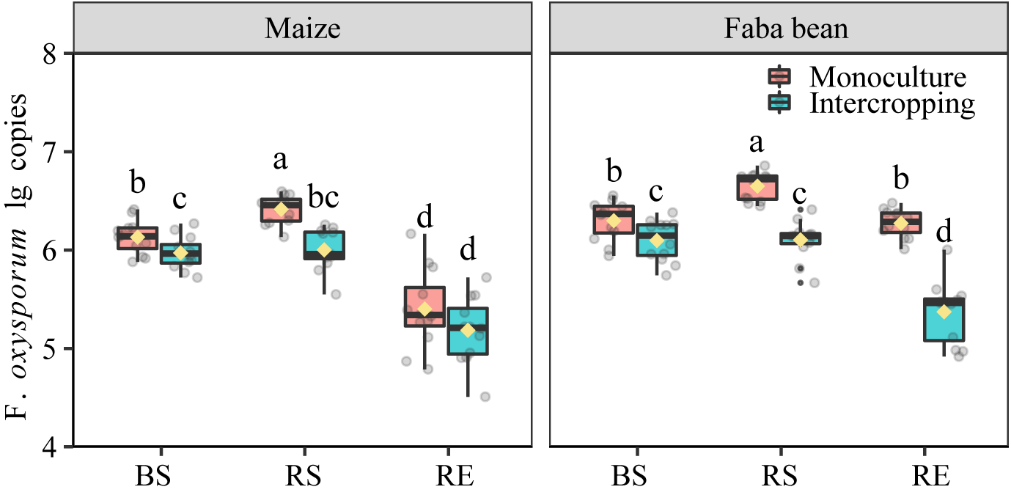


FIGURE S1 | The gene copies of *F. oxysporum* in different compartments (BS, RS, RE) of maize and faba bean in monoculture and intercropping. The top and bottom of each box represent the 25th and 75th percentiles, the horizontal line inside each box represents the 50th percentile/median and the whiskers represent the range of the points excluding outliers. Outliers are denoted as small black points outside whiskers. The yellow diamond symbols are the averages of the boxplots. Boxes with different letters indicate significant differences by Duncan’s multiple range test (*P* < 0.05). Monoculture, n = 12; intercropping, n = 12; BS, bulk soil; RS, rhizosphere soil; RE, root endosphere.


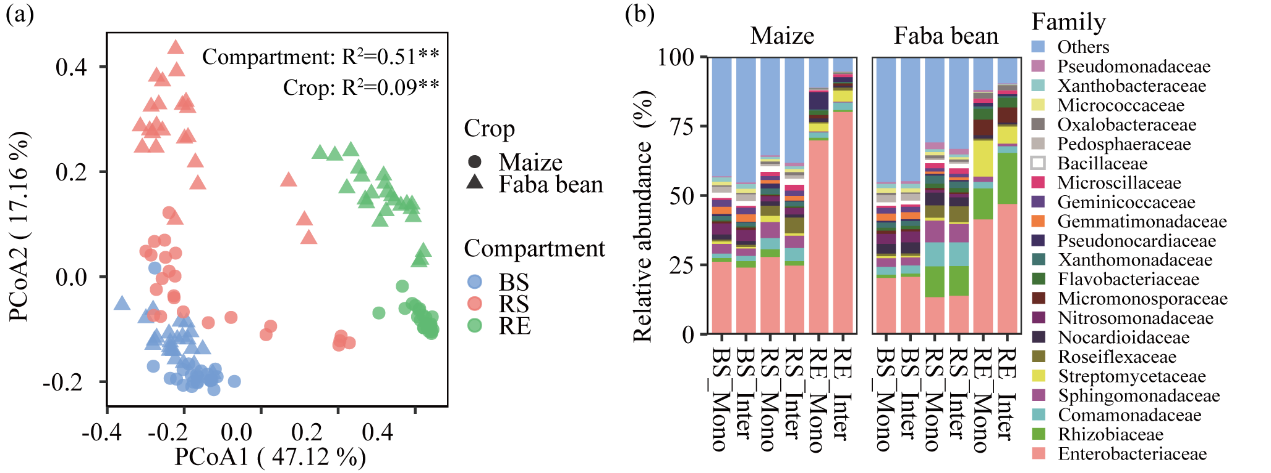


**FIGURE S2** **|** Principal coordinates analysis based on the Bray-Curtis distance showing that the bacteria of all samples is distinct from compartments and crop species (a), and the relative abundances of bacteria in BS, RS and RE of maize (left) and faba bean (right) in monoculture (Mono) and intercropping (Inter) at the family level (b). BS, bulk soil; RS, rhizosphere soil; RE, root endosphere; Mono, monoculture; Inter, intercropping; **, *P* < 0.01 by Permutational multivariate analysis of variance (PERMANOVA) using Adonis.


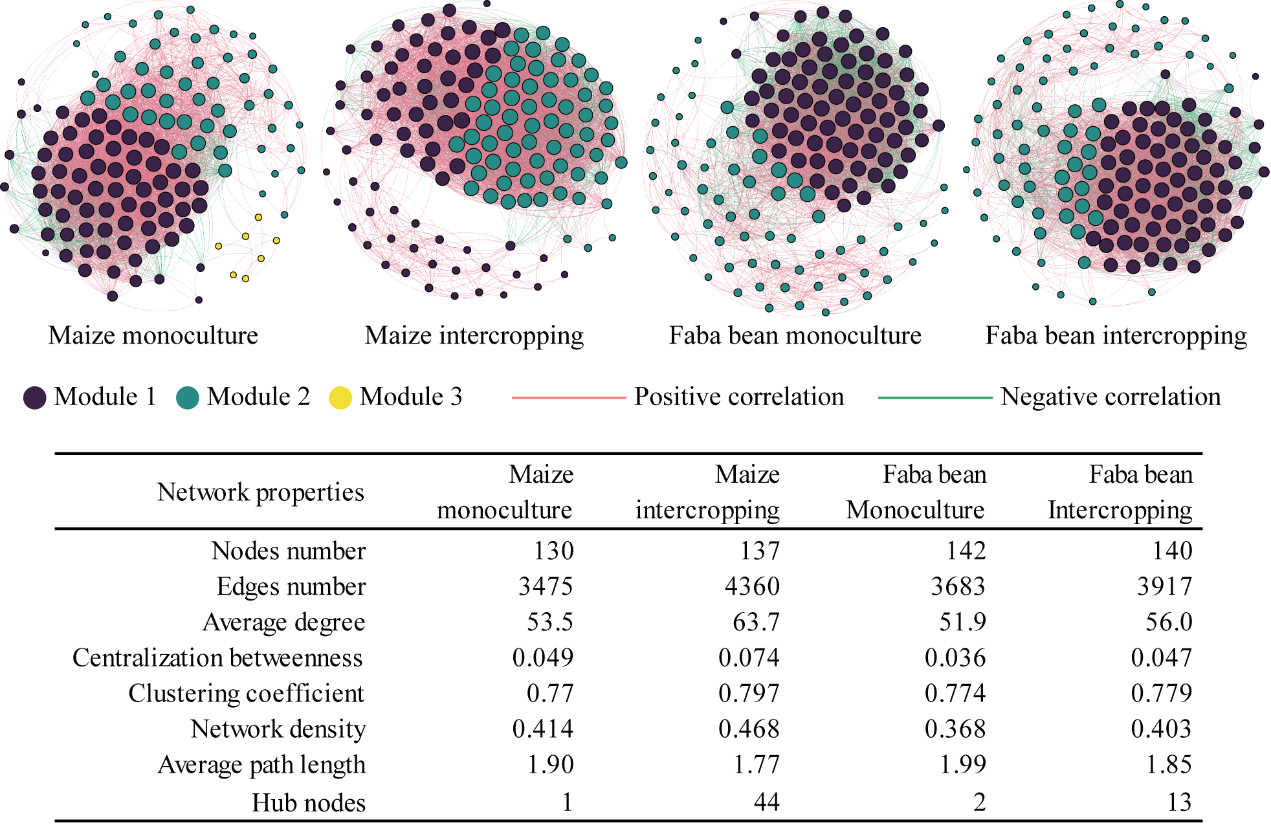


**FIGURE** **S3 |** Co-occurrence bacterial networks in monoculture and intercropping of maize and faba bean. Data are pooled across all compartments and fertilization treatments. The nodes are colored according to the modules in the network. The node size indicates the degree of connections. Edge colors represents positive (red) and negative (green) correlations. Lines indicate significant correlations between two nodes (r > 0.6 and *P* < 0.01). Black, green and yellow denote modules 1, 2 and 3.


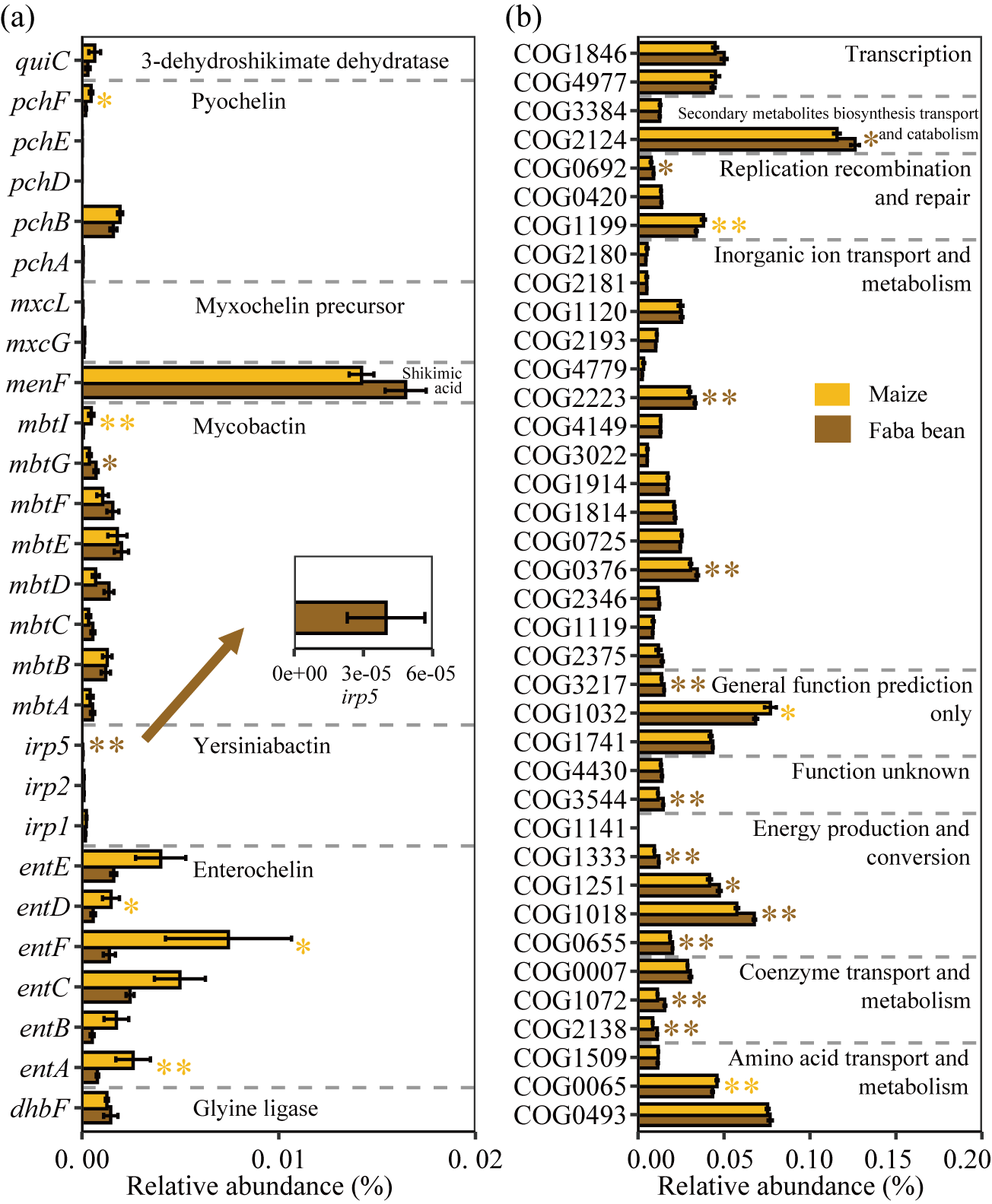


**FIGURE S4** **|** Relative abundances of nonribosomal peptide synthetase (NRPS) genes in rhizosphere soil of maize and faba bean based on metagenomic sequencing analysis (a). Relative abundances of COG categories related to iron metabolism in rhizosphere microbiome of maize and faba bean (b). Data (n = 6; mean ± S.E.) are from maize and faba bean rhizosphere. Golden asterisks indicate that the relative abundances of genes are significantly higher in maize than in faba bean. The brown asterisks are the opposite. *, *P* < 0.05; **, *P* < 0.01 significant differences are tested by Student’s t-test.


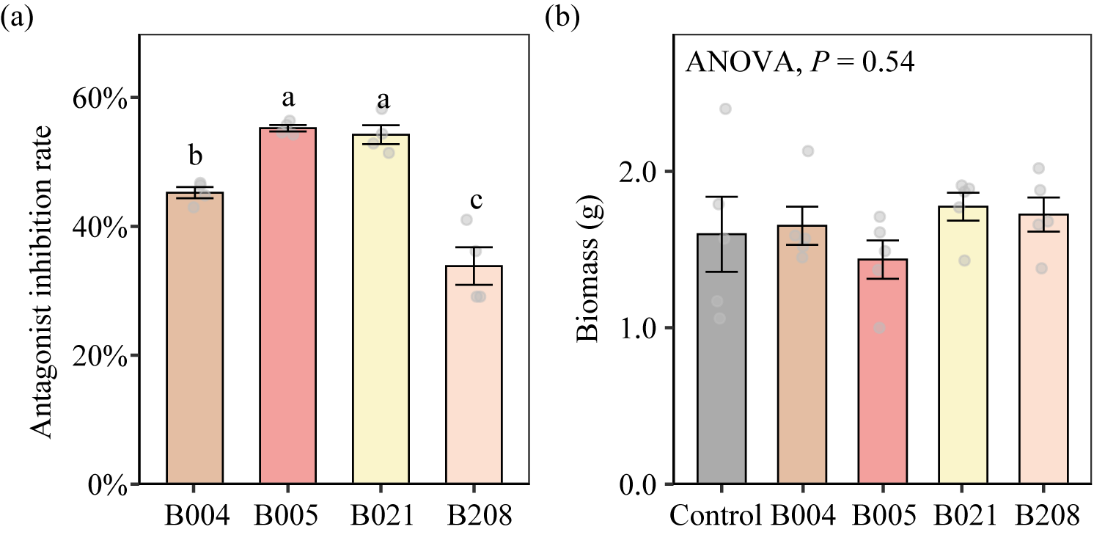


**FIGURE S5 |** Inhibition rates of the four isolates on FOF mycelial growth (a), and the biomass of faba bean in response to the inoculation of four antagonistic bacteria exposed to FOF (b). B004 and B021 are affiliated with *Pseudomonas chlororaphis*, and B005 and B208 are with *Bacillus megaterium* and *Bacillus subtilis*, respectively. Different letters indicate significant differences by Duncan’s multiple range test at *P* < 0.05. In panel (a), data (n = 4; mean ± S.E.) of the four isolates on FOF mycelial growth are presented. In panel (b), data (n = 5; mean ± S.E.) of the four isolates and the control (sterilized water) on faba bean biomass are presented in the presence of FOF.

**Supplementary tables**

**Table S1** Yields (kg ha^-1^) of faba bean and maize as affected by fertilization and planting pattern

| Fertilization | Faba bean |  | Maize |  |
| --- | --- | --- | --- | --- |
|  | Monoculture | Intercropping | Monoculture | Intercropping |
| P0N0 | 2675 ± 175 a | 2610 ± 197 b | 4900 ± 142 d | 6795 ± 92 b *** |
| P0N1 | 2403 ± 99 a | 2621 ± 168 b | 7342 ± 278 b | 7412 ± 800 b |
| P1N0 | 3041 ± 258 a | 3775 ± 183 a · | 5681 ± 307 c | 7719 ± 252 b ** |
| P1N1 | 3002 ± 186 a | 3715 ± 177 a * | 9419 ± 96 a | 11235±517 a · |
|  |  |  |  |  |
| ANOVA | F value | *P* value | F value | *P* value |
| Fertilization (F) | 56.77 | **< 0.001** | 9.50 | **0.011** |
| Planting pattern (P) | 33.32 | **< 0.001** | 13.03 | **0.007** |
| F × P | 3.39 | 0.074 | 3.11 | 0.089 |

Data are mean ± S.E. (n = 3). Values followed by the same lowercase letters are not significantly different among fertilization treatments within the same planting pattern at the 5% level according to Duncan’s multiple range test. Values followed by asterisks are significantly different between monoculture and intercropping within the same fertilization at the 5% level by Student's t-test. Bold text indicates significance level of *P* <0.05. ·, *P* < 0.1; *, *P* < 0.05; **, *P* < 0.01.

**Table S2** Effects of fertilization on partial land equivalent ratio (pLER) and land equivalent ratio (LER)

| Fertilization | pLER | | LER |
| --- | --- | --- | --- |
|  | Faba bean | Maize |  |
| P0N0 | 0.42 ± 0.02 a | 0.79 ± 0.03 a | 1.21 ± 0.04 ab |
| P0N1 | 0.47 ± 0.05 a | 0.57 ± 0.04 b | 1.05 ± 0.05 b |
| P1N0 | 0.54 ± 0.06 a | 0.78 ± 0.06 a | 1.32 ± 0.09 a |
| P1N1 | 0.53 ± 0.01 a | 0.68 ± 0.04 ab | 1.21 ± 0.04 ab |
|  |  |  |  |
| ANOVA |  |  |  |
| F value | 2.20 | 4.98 | 3.54 |
| *P* value | 0.17 | **0.03** | 0.07 |

Data are mean ± S.E. (n = 3). Values followed by the same lowercase letters are not significantly different among fertilization treatments within the same crop planting pattern at the 5% level according to Duncan’s multiple range test. Bold text indicates significance level of *P* <0.05.

**Table S3** Physicochemical properties of bulk soils in monoculture and intercropping of maize and faba bean fertilized with N and/or P

| Crop | Planting pattern | Fertilization | pH | TC  (g kg^-1^) | TN  (g kg^-1^) | DOC  (mg kg^-1^) | NH_4_^+^-N  (mg kg^-1^) | NO_3_^—^N  (mg kg^-1^) | AP  (mg kg^-1^) |
| --- | --- | --- | --- | --- | --- | --- | --- | --- | --- |
| Maize | Monoculture | P0N0 | 7.59 ± 0.04 (a) | 17.06 ± 0.34 (a) | 0.84 ± 0.03 (a) | 63.81 ± 6.61 (a) | 1.50 ± 0.11 (a) | 16.27 ± 0.83 (a) | 2.88 ± 0.18 (b) * |
|  |  | P0N1 | 7.57 ± 0.08 (a) | 17.29 ± 0.13 (a) | 0.89 ± 0.00 (a) | 74.35 ± 7.65 (a) | 1.58 ± 0.11 (a) | 22.49 ± 1.71 (a) * | 2.58 ± 0.51 (b) |
|  |  | P1N0 | 7.57 ± 0.08 (a) | 17.24 ± 0.30 (a) | 1.05 ± 0.13 (a) | 70.35 ± 1.20 (a) | 1.21 ± 0.17 (a) | 21.96 ± 1.66 (a) | 25.84 ± 2.7 (a) |
|  |  | P1N1 | 7.58 ± 0.03 (a) * | 17.78 ± 0.59 (a) | 0.94 ± 0.08 (a) | 72.75 ± 3.83 (a) | 1.43 ± 0.08 (a) | 22.52 ± 3.56 (a) | 19.54 ± 0.72 (a) |
|  | Intercropping | P0N0 | 7.50 ± 0.04 (A) | 17.09 ± 0.29 (A) | 0.90 ± 0.06 (A) | 69.97 ± 3.72 (A) | 1.70 ± 0.14 (A) | 17.63 ± 1.88 (A) | 3.72 ± 0.19 (B) * |
|  |  | P0N1 | 7.44 ± 0.06 (A) | 17.65 ± 0.70 (A) | 0.89 ± 0.04 (A) | 78.99 ± 7.29 (A) | 1.81 ± 0.14 (A) | 16.30 ± 1.34 (A) * | 4.28 ± 0.71 (B) |
|  |  | P1N0 | 7.42 ± 0.10 (A) | 17.90 ± 0.39 (A) | 1.01 ± 0.08 (A) | 72.72 ± 2.85 (A) | 1.49 ± 0.04 (A) | 18.76 ± 2.50 (A) | 27.72 ± 2.66 (A) |
|  |  | P1N1 | 7.33 ± 0.07 (A) * | 17.71 ± 0.84 (A) | 1.07 ± 0.03 (A) | 66.97 ± 8.38 (A) | 1.67 ± 0.23 (A) | 19.78 ± 0.80 (A) | 18.65 ± 1.89 (A) |
|  |  |  |  |  |  |  |  |  |  |
| Faba bean | Monoculture | P0N0 | 7.61 ± 0.05 (a) | 17.36 ± 0.22 (b) * | 1.04 ± 0.05 (a) * | 71.65 ± 9.01 (a) | 0.84 ± 0.16 (ab) | 13.66 ± 2.15 (a) | 2.77 ± 0.42 (b) |
|  |  | P0N1 | 7.66 ± 0.08 (a) | 17.70 ± 0.12 (ab) | 0.93 ± 0.08 (a) | 76.01 ± 10.1 (a) | 0.90 ± 0.14 (a) | 16.65 ± 1.78 (a) | 3.49 ± 0.52 (b) |
|  |  | P1N0 | 7.64 ± 0.10 (a) | 18.27 ± 0.30 (a) | 1.08 ± 0.04 (a) * | 84.81 ± 2.81 (a) | 0.51 ± 0.05 (b) | 17.22 ± 2.12 (a) · | 26.51 ± 1.72 (a) |
|  |  | P1N1 | 7.71 ± 0.06 (a) | 17.64 ± 0.20 (ab) | 0.94 ± 0.06 (a) | 80.98 ± 2.44 (a) | 0.53 ± 0.04 (b) | 19.08 ± 1.99 (a) | 28.3 ± 3.52 (a) |
|  | Intercropping | P0N0 | 7.59 ± 0.03 (A) | 18.27 ± 0.24 (A) * | 0.84 ± 0.02 (B) * | 73.75 ± 6.23 (A) | 0.63 ± 0.10 (A) | 17.33 ± 2.46 (A) | 4.47 ± 1.02 (C) |
|  |  | P0N1 | 7.51 ± 0.08 (A) | 17.69 ± 0.52 (A) | 0.95 ± 0.02 (A) | 73.02 ± 3.06 (A) | 0.64 ± 0.06 (A) | 20.51 ± 2.39 (A) | 3.03 ± 0.39 (C) |
|  |  | P1N0 | 7.51 ± 0.15 (A) | 17.85 ± 0.43 (A) | 0.90 ± 0.03 (AB) * | 77.39 ± 0.30 (A) | 0.48 ± 0.10 (A) | 24.97 ± 2.74 (A) · | 28.95 ± 2.93 (A) |
|  |  | P1N1 | 7.61 ± 0.04 (A) | 17.37 ± 0.30 (A) | 0.99 ± 0.04 (A) | 81.83 ± 1.69 (A) | 0.44 ± 0.01 (A) | 18.29 ± 0.90 (A) | 19.57 ± 1.80 (B) |
|  |  |  |  |  |  |  |  |  |  |
|  |  | Source of variation | *P* value | *P* value | *P* value | *P* value | *P* value | *P* value | *P* value |
| Maize | ANOVA | Fertilization (F) | 0.319 | **0.038** | 0.112 | 0.651 | 0.340 | 0.243 | **< 0.001** |
|  |  | Planting pattern (P) | **< 0.001** | 0.498 | 0.235 | 0.570 | **0.020** | 0.119 | 0.480 |
|  |  | F × P | 0.161 | 0.867 | 0.244 | 0.563 | 0.991 | 0.436 | 0.835 |
|  |  |  |  |  |  |  |  |  |  |
| Faba bean | ANOVA | Fertilization (F) | 0.505 | **0.009** | 0.743 | 0.392 | **0.035** | 0.208 | **< 0.001** |
|  |  | Planting pattern (P) | **0.001** | 0.809 | **0.023** | 0.565 | **0.014** | **0.021** | 0.351 |
|  |  | F × P | 0.167 | 0.182 | **0.027** | 0.708 | 0.354 | 0.207 | 0.051 |

Data are mean ± S.E. (n = 3). Values followed by the same lowercase letters are not significantly different among fertilization treatments within the same crop planting pattern at the 5% level by Duncan’s multiple range test. Values followed by asterisks are significantly different between monoculture and intercropping within the same fertilization at the 5% level according to Student's t-test. Bold text indicates significance level of *P* <0.05.

**Table S4** Gene copies of *F. oxysporum* as affected by fertilization and planting pattern in bulk soil, rhizosphere and root endosphere of faba bean and maize

| Crop | Planting pattern | Bulk soil | Rhizosphere soil | Root endosphere |
| --- | --- | --- | --- | --- |
| Faba bean | Monoculture | 6.30 ± 0.06 b | 6.65 ± 0.04 a | 6.27 ± 0.04 b |
|  | Intercropping | 6.10 ± 0.06 a * | 6.10 ± 0.06 a *** | 5.37 ± 0.09 b *** |
| Maize | Monoculture | 6.13 ± 0.05 b | 6.41 ± 0.04 a | 5.40 ± 0.12 c |
|  | Intercropping | 5.97 ± 0.05 a * | 6.00 ± 0.06 a *** | 5.18 ± 0.10 b |
|  |  |  |  |  |
| ANOVA | Source of variation | *P* value | *P* value | *P* value |
| Faba bean | Fertilization (F) | 1.12E-01 | 9.53E-01 | 6.85E-01 |
|  | Planting pattern (P) | **1.93E-04** | **1.80E-05** | **2.29E-05** |
|  | F × P | 1.38E-01 | **2.02E-02** | 2.54E-01 |
|  |  |  |  |  |
| Maize | Fertilization (F) | 5.31E-02 | 2.57E-01 | 8.04E-01 |
|  | Planting pattern (P) | **1.62E-02** | **1.80E-04** | 1.44E-01 |
|  | F × P | 7.02E-02 | 8.06E-02 | 5.97E-01 |

Data are mean ± S.E. (n = 3). Values followed by the same lowercase letters are not significantly different among fertilization treatments within the same crop planting pattern at the 5% level by Duncan’s multiple range test; values followed by asterisks are significantly different between monoculture and intercropping within the same fertilization at the 5% level according to Student's t-test. Bold text indicates significance level of *P* <0.05. *, *P* < 0.05; ***, *P* < 0.001.

**Table S5** Two-way ANOVA of the effects of planting pattern and fertilization treatments on bacterial α diversity in bulk soil (BS), rhizosphere (RS) and root endosphere (RE) of maize and faba bean

| Alpha diversity | Crop | Source of variation | Df | BS | | RS | | RE | |
| --- | --- | --- | --- | --- | --- | --- | --- | --- | --- |
|  |  |  |  | *F* value | *P* | *F* value | *P* | *F* value | *P* |
| Observed OTUs | Maize | Planting pattern (P) | 1 | 0.044 | 0.836 | 3.985 | **0.063** | 0.941 | 0.347 |
|  |  | Fertilization (F) | 3 | 0.797 | 0.513 | 0.459 | 0.714 | 0.812 | 0.506 |
|  |  | P × F | 3 | 0.229 | 0.875 | 0.924 | 0.452 | 1.156 | 0.357 |
|  |  |  |  |  |  |  |  |  |  |
|  | Faba bean | Planting pattern (P) | 1 | 0.087 | 0.772 | 3.549 | **0.078** | 0.038 | 0.848 |
|  |  | Fertilization (F) | 3 | 1.330 | 0.299 | 0.214 | 0.885 | 0.601 | 0.623 |
|  |  | P × F | 3 | 0.933 | 0.447 | 0.974 | 0.430 | 0.288 | 0.834 |
|  |  |  |  |  |  |  |  |  |  |
| Shannon index | Maize | Planting pattern (P) | 1 | 0.192 | 0.667 | 3.513 | **0.079** | 1.757 | 0.204 |
|  |  | Fertilization (F) | 3 | 1.325 | 0.301 | 1.065 | 0.392 | 4.209 | **0.023** |
|  |  | P × F | 3 | 0.328 | 0.805 | 1.125 | 0.369 | 3.211 | **0.051** |
|  |  |  |  |  |  |  |  |  |  |
|  | Faba bean | Planting pattern (P) | 1 | 1.066 | 0.317 | 0.836 | 0.374 | 0.256 | 0.620 |
|  |  | Fertilization (F) | 3 | 0.678 | 0.578 | 0.353 | 0.788 | 0.227 | 0.876 |
|  |  | P × F | 3 | 0.544 | 0.659 | 0.773 | 0.526 | 0.959 | 0.436 |

Bold text indicates significance level of *P* <0.1.

**Table S6** Effects of planting pattern and fertilization treatments on the abundant bacterial families in bulk soil (BS), rhizosphere (RS) and root endosphere (RE) of maize and faba bean

| Family | Planting pattern | | | | | | Fertilization | | | | | |
| --- | --- | --- | --- | --- | --- | --- | --- | --- | --- | --- | --- | --- |
|  | Maize | | | Faba bean | | | Maize | | | Faba bean | | |
|  | BS | RS | RE | BS | RS | RE | BS | RS | RE | BS | RS | RE |
| *Enterobacteriaceae* | 0.843 | 0.713 | **0.024** | 0.887 | 0.551 | 0.198 | 0.210 | **0.042** | 0.814 | 0.560 | 0.145 | 0.381 |
| *Rhizobiaceae* | 0.887 | **0.039** | 0.128 | 0.977 | 0.443 | 0.319 | 0.738 | 0.246 | 0.511 | 0.530 | 0.472 | 0.126 |
| *Comamonadaceae* | 0.291 | 0.319 | 0.219 | 0.755 | 0.630 | 0.478 | **0.018** | 0.071 | 0.460 | 0.200 | **0.043** | 0.079 |
| *Sphingomonadaceae* | **<0.001** | **0.028** | 1.000 | 0.443 | 0.347 | 0.052 | 0.416 | 0.062 | 0.778 | 0.940 | 0.862 | 0.050 |
| *Streptomycetaceae* | **<0.001** | **<0.001** | **0.028** | **0.004** | **0.039** | **0.021** | 0.059 | 0.387 | 0.536 | 0.950 | 0.443 | 0.935 |
| *Roseiflexaceae* | 0.291 | 0.219 | 0.068 | 0.977 | 0.799 | 0.630 | 0.538 | 0.603 | 0.878 | 0.980 | 0.992 | 0.855 |
| *Nocardioidaceae* | **0.005** | 0.319 | **0.024** | 0.551 | 0.347 | **0.003** | 0.108 | 0.364 | 0.654 | **<0.001** | 0.109 | 0.098 |
| *Nitrosomonadaceae* | 0.977 | 0.114 | 0.488 | 0.590 | 0.219 | 0.402 | 0.691 | 0.057 | 0.630 | 0.300 | 0.502 | 0.265 |
| *Micromonosporaceae* | 0.160 | 0.219 | 0.410 | **<0.001** | 0.219 | 0.478 | 0.059 | 0.313 | 0.808 | 0.180 | 0.577 | **0.013** |
| *Flavobacteriaceae* | **0.033** | 0.347 | 0.219 | 0.590 | 0.932 | 0.755 | 0.500 | 0.900 | 0.783 | 0.330 | 0.951 | 0.512 |
| *Xanthomonadaceae* | 0.242 | 0.755 | 0.443 | 0.887 | 0.266 | 0.671 | 0.077 | 0.146 | 0.596 | 0.420 | **0.004** | 0.170 |
| *Pseudonocardiaceae* | **<0.001** | **<0.001** | **<0.001** | 0.799 | 0.843 | 0.068 | 0.950 | 0.854 | 0.797 | 0.100 | 0.147 | 0.156 |
| *Gemmatimonadaceae* | 0.799 | **0.033** | 0.537 | 0.887 | 0.590 | 0.192 | 0.218 | 0.267 | 0.396 | 0.420 | 0.960 | 0.407 |
| *Geminicoccaceae* | 0.443 | 0.143 | 0.630 | 0.799 | 0.843 | **0.026** | 0.581 | 0.949 | 0.577 | 0.160 | 0.712 | **0.033** |
| *Microscillaceae* | 0.410 | 0.713 | 0.178 | 0.713 | 0.799 | 0.932 | 0.071 | 0.334 | 0.976 | 0.880 | 0.574 | 0.117 |
| *Bacillaceae* | 0.378 | 0.713 | 0.799 | 0.078 | 0.128 | 0.141 | 0.450 | 0.482 | 0.331 | 0.600 | 0.767 | 0.667 |
| *Pedosphaeraceae* | 0.378 | 0.052 | 0.069 | 0.514 | 0.977 | 0.399 | 0.846 | 0.207 | 0.847 | 0.840 | 0.793 | 0.842 |
| *Oxalobacteraceae* | 0.843 | 0.143 | 0.160 | **0.005** | 0.068 | 0.799 | 0.069 | **0.019** | 0.449 | 0.540 | 0.114 | 0.065 |
| *Micrococcaceae* | 0.160 | 0.755 | 0.378 | 0.671 | 0.160 | 0.478 | 0.116 | 0.135 | 0.446 | 0.250 | 0.808 | 0.125 |
| *Xanthobacteraceae* | 0.514 | 0.128 | 0.242 | 0.671 | 0.551 | 0.347 | 0.967 | 0.106 | 0.098 | 0.380 | 0.976 | 0.116 |
| *Pseudomonadaceae* | **0.020** | **0.012** | 0.799 | **0.039** | 0.291 | 0.101 | 0.831 | 0.307 | 0.904 | 0.620 | 0.521 | 0.743 |
| Others | **0.045** | 0.347 | 0.242 | 0.514 | 0.347 | 0.101 | 0.405 | 0.078 | 0.500 | 0.680 | 0.837 | 0.387 |

Bold text of different abundant families on planting pattern and fertilization indicates significant differences as defined by Student’s t-test and ANOVA, respectively, in each compartment of maize and faba bean.

**Table S7** Topological properties of empirical and random bacterial networks in the monoculture and intercropping of maize and faba bean

| Network type | Network properties | Maize monoculture | Maize intercropping | Faba bean monoculture | Faba bean intercropping |
| --- | --- | --- | --- | --- | --- |
| Empirical network | Module number | 3 | 2 | 2 | 2 |
|  | Positive proportion | 87.7% | 86.9% | 70.6% | 76.3% |
|  | Centralization degree | 0.252 | 0.253 | 0.249 | 0.245 |
|  | Network diameter | 6 | 6 | 5 | 5 |
|  | Modularity | 0.153 | 0.143 | 0.167 | 0.159 |
|  |  |  |  |  |  |
| Random network | Clustering coefficient | 0.414 | 0.467 | 0.367 | 0.402 |
|  | Average path length | 1.59 | 1.53 | 1.63 | 1.60 |
|  | Network diameter | 2 | 2 | 2 | 2 |
|  | Network density | 0.41 | 0.47 | 0.37 | 0.40 |
|  | Modularity | 0.0584 | 0.0475 | 0.064± | 0.0576 |

**Table S8** Hub nodes in monoculture and intercropping of maize and faba bean

| OTU ID | Degree | Affiliation | Number | Abundance | Family | Genus |
| --- | --- | --- | --- | --- | --- | --- |
| OTU1774 | 86 | MMO | 1 | 0.20% | *Bacillaceae* | *Bacillus* |
| OTU2362 | 98 | MIN | 44 | 0.35% | *Nitrosomonadaceae* | *Ellin6067* |
| OTU918 | 98 | MIN |  | 0.36% | *norank_o__norank_c__AKAU4049* | *norank_f__norank_o__norank_c__AKAU4049* |
| OTU2624 | 97 | MIN |  | 0.18% | *Xanthobacteraceae* | *Pseudolabrys* |
| OTU302 | 97 | MIN |  | 0.11% | *Geminicoccaceae* | *norank_f__Geminicoccaceae* |
| OTU2529 | 96 | MIN |  | 0.19% | *Geminicoccaceae* | *norank_f__Geminicoccaceae* |
| OTU2776 | 96 | MIN |  | 0.33% | *SC-I-84* | *norank_f__SC-I-84* |
| OTU3235 | 96 | MIN |  | 0.14% | *norank_o__S085* | *norank_f__norank_o__S085* |
| OTU625 | 96 | MIN |  | 0.35% | *Geminicoccaceae* | *norank_f__Geminicoccaceae* |
| OTU12 | 95 | MIN |  | 0.23% | *Sphingomonadaceae* | *Sphingomonas* |
| OTU3185 | 95 | MIN |  | 0.11% | *Sutterellaceae* | *norank_f__Sutterellaceae* |
| OTU832 | 95 | MIN |  | 0.13% | *Gemmatimonadaceae* | *norank_f__Gemmatimonadaceae* |
| OTU157 | 94 | MIN |  | 0.19% | *Solirubrobacteraceae* | *Solirubrobacter* |
| OTU1944 | 94 | MIN |  | 0.53% | *Sphingomonadaceae* | *Sphingomonas* |
| OTU2067 | 94 | MIN |  | 0.20% | *Nitrosomonadaceae* | *Ellin6067* |
| OTU2131 | 94 | MIN |  | 0.13% | *Gaiellaceae* | *Gaiella* |
| OTU2132 | 94 | MIN |  | 0.10% | *norank_o__Rhizobiales* | *norank_f__norank_o__Rhizobiales* |
| OTU2240 | 94 | MIN |  | 0.19% | *67-14* | *norank_f__67-14* |
| OTU1286 | 93 | MIN |  | 0.39% | *norank_o__PLTA13* | *norank_f__norank_o__PLTA13* |
| OTU3182 | 93 | MIN |  | 0.11% | *norank_o__Azospirillales* | *norank_f__norank_o__Azospirillales* |
| OTU3360 | 93 | MIN |  | 0.17% | *norank_o__norank_c__Thermoleophilia* | *norank_f__norank_o__norank_c__Thermoleophilia* |
| OTU862 | 93 | MIN |  | 0.11% | *Pedosphaeraceae* | *ADurb.Bin063-1* |
| OTU1395 | 92 | MIN |  | 0.42% | *Geminicoccaceae* | *norank_f__Geminicoccaceae* |
| OTU1430 | 92 | MIN |  | 0.12% | *Hyphomicrobiaceae* | *norank_f__Hyphomicrobiaceae* |
| OTU1871 | 92 | MIN |  | 0.14% | *Gemmatimonadaceae* | *norank_f__Gemmatimonadaceae* |
| OTU1913 | 92 | MIN |  | 0.13% | *Gemmatimonadaceae* | *norank_f__Gemmatimonadaceae* |
| OTU2203 | 92 | MIN |  | 0.18% | *Bacillaceae* | *Bacillus* |
| OTU282 | 92 | MIN |  | 0.16% | *Xanthobacteraceae* | *norank_f__Xanthobacteraceae* |
| OTU327 | 92 | MIN |  | 0.18% | *67-14* | *norank_f__67-14* |
| OTU1458 | 91 | MIN |  | 0.19% | *Bacillaceae* | *Bacillus* |
| OTU2121 | 91 | MIN |  | 0.19% | *Dongiaceae* | *Dongia* |
| OTU3090 | 90 | MIN |  | 0.20% | *Beijerinckiaceae* | *Microvirga* |
| OTU413 | 89 | MIN |  | 0.11% | *Bacillaceae* | *Bacillus* |
| OTU472 | 89 | MIN |  | 0.13% | *67-14* | *norank_f__67-14* |
| OTU595 | 89 | MIN |  | 0.10% | *Thermoanaerobaculaceae* | *Subgroup_10* |
| OTU1059 | 88 | MIN |  | 0.66% | *Micrococcaceae* | *Arthrobacter* |
| OTU923 | 88 | MIN |  | 0.17% | *Entotheonellaceae* | *norank_f__Entotheonellaceae* |
| OTU2061 | 87 | MIN |  | 0.51% | *Gemmatimonadaceae* | *norank_f__Gemmatimonadaceae* |
| OTU2373 | 87 | MIN |  | 0.15% | *Nitrosomonadaceae* | *MND1* |
| OTU3258 | 87 | MIN |  | 0.15% | *norank_o__norank_c__TK10* | *norank_f__norank_o__norank_c__TK10* |
| OTU456 | 87 | MIN |  | 0.25% | *Steroidobacteraceae* | *norank_f__Steroidobacteraceae* |
| OTU1514 | 86 | MIN |  | 0.14% | *norank_o__norank_c__norank_p__NB1-j* | *norank_f__norank_o__norank_c__norank_p__NB1-j* |
| OTU1931 | 86 | MIN |  | 0.18% | *Nitrosomonadaceae* | *MND1* |
| OTU3062 | 86 | MIN |  | 0.42% | *Xanthomonadaceae* | *Lysobacter* |
| OTU922 | 86 | MIN |  | 0.24% | *Nitrospiraceae* | *Nitrospira* |
| OTU2362 | 87 | FMO | 2 | 0.28% | *Nitrosomonadaceae* | *Ellin6067* |
| OTU2230 | 86 | FMO |  | 0.36% | *Geodermatophilaceae* | *Blastococcus* |
| OTU1720 | 90 | FIN | 13 | 2.07% | *Streptomycetaceae* | *Streptomyces* |
| OTU2198 | 89 | FIN |  | 0.15% | *Xanthomonadaceae* | *unclassified_f__Xanthomonadaceae* |
| OTU282 | 89 | FIN |  | 0.19% | *Xanthobacteraceae* | *norank_f__Xanthobacteraceae* |
| OTU1512 | 88 | FIN |  | 0.10% | *Micromonosporaceae* | *unclassified_f__Micromonosporaceae* |
| OTU1942 | 88 | FIN |  | 0.39% | *Sphingomonadaceae* | *Sphingomonas* |
| OTU1899 | 87 | FIN |  | 0.22% | *unclassified_p__Patescibacteria* | *unclassified_p__Patescibacteria* |
| OTU3360 | 87 | FIN |  | 0.13% | *norank_o__norank_c__Thermoleophilia* | *norank_f__norank_o__norank_c__Thermoleophilia* |
| OTU1522 | 86 | FIN |  | 0.67% | *Thermomonosporaceae* | *Actinocorallia* |
| OTU1774 | 86 | FIN |  | 0.15% | *Bacillaceae* | *Bacillus* |
| OTU1944 | 86 | FIN |  | 0.49% | *Sphingomonadaceae* | *Sphingomonas* |
| OTU2132 | 86 | FIN |  | 0.10% | *norank_o__Rhizobiales* | *norank_f__norank_o__Rhizobiales* |
| OTU2776 | 86 | FIN |  | 0.27% | *SC-I-84* | *norank_f__SC-I-84* |
| OTU472 | 86 | FIN |  | 0.11% | *67-14* | *norank_f__67-14* |

Here, OTUs with degree > 85 were identified as potential keystone taxa.

**Table S9** Isolates from bulk soil and sequenced by 16S rRNA

| Strain label | Closest relative | Genus | Phylum |
| --- | --- | --- | --- |
| B001 | *Pseudomonas* sp. | *Pseudomonas* | *Proteobacteria* |
| B002 | *Bacillus subtilis subsp. Subtilis* | *Bacillus* | *Firmicutes* |
| B003 | *Pseudomonas syringae* | *Pseudomonas* | *Proteobacteria* |
| B004 | *Pseudomonas chlororaphis* | *Pseudomonas* | *Proteobacteria* |
| B005 | *Bacillus* sp. | *Bacillus* | *Firmicutes* |
| B006 | *Pseudomonas laurylsulfativorans* | *Pseudomonas* | *Proteobacteria* |
| B007 | *Pseudomonas mandelii* | *Pseudomonas* | *Proteobacteria* |
| B008 | *Klebsiella* sp. | *Klebsiella* | *Proteobacteria* |
| B009 | *Klebsiella variicola* | *Klebsiella* | *Proteobacteria* |
| B010 | *Klebsiella variicola* | *Klebsiella* | *Proteobacteria* |
| B011 | *Bacillus megaterium* | *Bacillus* | *Firmicutes* |
| B012 | *Klebsiella* sp. | *Klebsiella* | *Proteobacteria* |
| B013 | *Klebsiella variicola* | *Klebsiella* | *Proteobacteria* |
| B014 | *Escherichia* sp. *76.3* | *Escherichia* | *Proteobacteria* |
| B015 | *Bacillus megaterium* | *Bacillus* | *Firmicutes* |
| B016 | *Escherichia coli* | *Escherichia* | *Proteobacteria* |
| B017 | *Escherichia coli* | *Escherichia* | *Proteobacteria* |
| B018 | *Pseudomonas aeruginosa* | *Pseudomonas* | *Proteobacteria* |
| B019 | *Pseudomonas plecoglossicida* | *Pseudomonas* | *Proteobacteria* |
| B020 | *Escherichia* sp. *76.3* | *Escherichia* | *Proteobacteria* |
| B021 | *Pseudomonas* sp. | *Pseudomonas* | *Proteobacteria* |
| B022 | *Escherichia coli* | *Escherichia* | *Proteobacteria* |
| B023 | *Escherichia coli* | *Escherichia* | *Proteobacteria* |
| B024 | *Acinetobacter calcoaceticus* | *Acinetobacter* | *Proteobacteria* |
| B025 | *Escherichia coli* | *Escherichia* | *Proteobacteria* |
| B026 | *Escherichia coli* | *Escherichia* | *Proteobacteria* |
| B027 | *Escherichia coli* | *Escherichia* | *Proteobacteria* |
| B028 | *Escherichia coli* | *Escherichia* | *Proteobacteria* |
| B029 | *Escherichia coli* | *Escherichia* | *Proteobacteria* |
| B030 | *Escherichia coli* | *Escherichia* | *Proteobacteria* |
| B031 | *Escherichia coli* | *Escherichia* | *Proteobacteria* |
| B032 | *Pseudomonas mandelii* | *Pseudomonas* | *Proteobacteria* |
| B033 | *Escherichia* sp. *76.3* | *Escherichia* | *Proteobacteria* |
| B034 | *Escherichia coli* | *Escherichia* | *Proteobacteria* |
| B035 | *Escherichia coli* | *Escherichia* | *Proteobacteria* |
| B036 | *Pseudomonas* sp. *JSPB3* | *Pseudomonas* | *Proteobacteria* |
| B037 | *Escherichia coli* | *Escherichia* | *Proteobacteria* |
| B038 | *Pseudomonas* sp. *JSPB3* | *Pseudomonas* | *Proteobacteria* |
| B039 | *Pseudomonas* sp. | *Pseudomonas* | *Proteobacteria* |
| B040 | *Pseudomonas corrugata* | *Pseudomonas* | *Proteobacteria* |
| B041 | *Bacillus megaterium* | *Bacillus* | *Firmicutes* |
| B042 | *Pseudomonas donghuensis* | *Pseudomonas* | *Proteobacteria* |
| B043 | *Pseudomonas donghuensis* | *Pseudomonas* | *Proteobacteria* |
| B044 | *Escherichia coli* | *Escherichia* | *Proteobacteria* |
| B045 | *Escherichia coli* | *Escherichia* | *Proteobacteria* |
| B046 | *Pseudomonas donghuensis* | *Pseudomonas* | *Proteobacteria* |
| B047 | *Pseudomonas frederiksbergensis* | *Pseudomonas* | *Proteobacteria* |
| B048 | *Escherichia* sp. *76.3* | *Escherichia* | *Proteobacteria* |
| B049 | *Chitinophaga ginsengisegetis* | *Chitinophaga* | *Bacteroidota* |
| B050 | *Bacillus aryabhattai* | *Bacillus* | *Firmicutes* |
| B051 | *Aminobacter aminovorans* | *Aminobacter* |  |
| B052 | *Lysobacter antibioticus* | *Lysobacter* | *Proteobacteria* |
| B053 | *Lysobacter* sp. | *Lysobacter* | *Proteobacteria* |
| B054 | *Chitinophaga ginsengisegetis* | *Chitinophaga* | *Bacteroidota* |
| B129 | *Pseudomonas frederiksbergensis* | *Pseudomonas* | *Proteobacteria* |
| B130 | *Pseudomonas* sp. | *Pseudomonas* | *Proteobacteria* |
| B131 | *Acinetobacter calcoaceticus* | *Acinetobacter* | *Proteobacteria* |
| B132 | *Ochrobactrum* sp. | *Ochrobactrum* | *Proteobacteria* |
| B133 | *Pseudomonas putida* | *Pseudomonas* | *Proteobacteria* |
| B134 | *Pseudomonas frederiksbergensis* | *Pseudomonas* | *Proteobacteria* |
| B135 | *Pseudomonas frederiksbergensis* | *Pseudomonas* | *Proteobacteria* |
| B136 | *Pseudomonas frederiksbergensis* | *Pseudomonas* | *Proteobacteria* |
| B137 | *Pseudomonas frederiksbergensis* | *Pseudomonas* | *Proteobacteria* |
| B138 | *Bacillus aryabhattai* | *Bacillus* | *Firmicutes* |
| B139 | *Pseudomonas mandelii* | *Pseudomonas* | *Proteobacteria* |
| B140 | *Bacillus ginsengisoli* | *Bacillus* | *Firmicutes* |
| B141 | *Bacillus megaterium* | *Bacillus* | *Firmicutes* |
| B142 | *Pseudomonas syringae* | *Pseudomonas* | *Proteobacteria* |
| B143 | *Pseudomonas mandelii* | *Pseudomonas* | *Proteobacteria* |
| B144 | *Pseudomonas frederiksbergensis* | *Pseudomonas* | *Proteobacteria* |
| B145 | *Bacillus aryabhattai* | *Bacillus* | *Firmicutes* |
| B146 | *Pseudomonas* sp. | *Pseudomonas* | *Proteobacteria* |
| B147 | *Pseudomonas* sp. | *Pseudomonas* | *Proteobacteria* |
| B148 | *Acinetobacter calcoaceticus* | *Acinetobacter* | *Proteobacteria* |
| B149 | *Acinetobacter* sp. | *Acinetobacter* | *Proteobacteria* |
| B150 | *Acinetobacter calcoaceticus* | *Acinetobacter* | *Proteobacteria* |
| B151 | *Pseudomonas frederiksbergensis* | *Pseudomonas* | *Proteobacteria* |
| B152 | *Acinetobacter calcoaceticus* | *Acinetobacter* | *Proteobacteria* |
| B165 | *Bacillus aryabhattai* | *Bacillus* | *Firmicutes* |
| B166 | *Pseudomonas plecoglossicida* | *Pseudomonas* | *Proteobacteria* |
| B167 | *Pantoea dispersa* | *Pantoea* | *Proteobacteria* |
| B168 | *Pantoea* sp. | *Pantoea* | *Proteobacteria* |
| B169 | *Pantoea* sp. | *Pantoea* | *Proteobacteria* |
| B170 | *Agrobacterium tumefaciens* | *Agrobacterium* |  |
| B204 | *Paenarthrobacter nitroguajacolicus* | *Paenarthrobacter* |  |
| B205 | *Stenotrophomonas rhizophila* | *Stenotrophomonas* | *Proteobacteria* |
| B206 | *Stenotrophomonas rhizophila* | *Stenotrophomonas* | *Proteobacteria* |
| B207 | *Pantoea dispersa* | *Pantoea* | *Proteobacteria* |
| B208 | *Bacillus subtilis* | *Bacillus* | *Firmicutes* |
| B209 | *Pseudomonas* | *Pseudomonas* | *Proteobacteria* |
| B210 | *Pseudomonas* | *Pseudomonas* | *Proteobacteria* |
| B211 | *Pseudomonas chlororaphis* | *Pseudomonas* | *Proteobacteria* |
| B212 | *Pseudomonas* | *Pseudomonas* | *Proteobacteria* |
| B213 | *Bacillus subtilis* | *Bacillus* | *Firmicutes* |
| B215 | *Enterobacter* sp. | *Enterobacter* |  |

**References**

Bastian, M., Heymann, S., and Jacomy, M. (2009). Gephi : An open source software for exploring and manipulating networks. *ICWSM* 8, 361-362. doi: 10.13140/2.1.1341.1520

Beckers, B., Op De Beeck, M., Weyens, N., Boerjan, W., and Vangronsveld, J. (2017). Structural variability and niche differentiation in the rhizosphere and endosphere bacterial microbiome of field-grown poplar trees. *Microbiome* 5, 25. doi: 10.1186/s40168-017-0241-2

Benjamini, Y., and Hochberg, Y. (1995). Controlling the false discovery rate: A practical and powerful approach to multiple testing. *J. R. Stat. Soc. B.* 57, 289-300. doi: 10.1111/j.2517-6161.1995.tb02031.x

Bolger, A.M., Lohse, M., and Usadel, B. (2014). Trimmomatic: A flexible trimmer for illumina sequence data. *Bioinformatics* 30, 2114-2120. doi: 10.1093/bioinformatics/btu170

Csardi, G., and Nepusz, T. (2006). The igraph software package for complex network research. *Inter. J. Complex Syst.* 1695, 1-9. Available online at: http://igraph.org

Edgar, R.C. (2013). Uparse: Highly accurate otu sequences from microbial amplicon reads. *Nat. Methods* 10, 996-1000. doi: 10.1038/Nmeth.2604

Erdos, P. (1970). On sets of distances of n points. *The American Mathematical Monthly* 77, 165–169. doi: 10.2307/2316209

Gardes, M., and Bruns, T.D. (1993). Its primers with enhanced specificity for basidiomycetes-application to the identification of mycorrhizae and rusts. *Mol. Ecol.* 2, 113-118. doi: 10.1111/j.1365-294x.1993.tb00005.x

Hartman, K., van der Heijden, M.G.A., Wittwer, R.A., Banerjee, S., Walser, J.C., and Schlaeppi, K. (2018). Cropping practices manipulate abundance patterns of root and soil microbiome members paving the way to smart farming. *Microbiome* 6. doi: 10.1186/s40168-017-0389-9

Hassani, M.A., Durán, P., and Hacquard, S. (2018). Microbial interactions within the plant holobiont. *Microbiome* 6, 58. doi: 10.1186/s40168-018-0445-0

Jiao, S., Yang, Y., Xu, Y., Zhang, J., and Lu, Y. (2020). Balance between community assembly processes mediates species coexistence in agricultural soil microbiomes across eastern china. *ISME J.* 14, 202-216. doi: 10.1038/s41396-019-0522-9

Jones, D.L., and Willett, V.B. (2006). Experimental evaluation of methods to quantify dissolved organic nitrogen (DON) and dissolved organic carbon (DOC) in soil. *Soil Biol. Biochem.* 38, 991-999. doi: 10.1016/j.soilbio.2005.08.012

Klaedtke, S., Jacques, M.A., Raggi, L., Preveaux, A., Bonneau, S., Negri, V. et al. (2016). Terroir is a key driver of seed-associated microbial assemblages. *Environ. Microbiol.* 18, 1792-1804. doi: 10.1111/1462-2920.12977

Langfelder, P., and Horvath, S. (2012). Fastrfunctions for robust correlations and hierarchical clustering. *J. Stat. Softw.* 46. doi: 10.18637/jss.v046.i11

Liao, D., Zhang, C.C., Lambers, H., and Zhang, F.S. (2021). Changes in soil phosphorus fractions in response to long-term phosphate fertilization under sole cropping and intercropping of maize and faba bean on a calcareous soil. *Plant Soil* 463, 589-600. doi: 10.1007/s11104-021-04915-y

Lievens, B., Brouwer, M., Vanachter, A.C., Levesque, C.A., Cammue, B.P., and Thomma, B.P. (2005). Quantitative assessment of phytopathogenic fungi in various substrates using a DNA macroarray. *Environ. Microbiol.* 7, 1698-1710. doi: 10.1111/j.1462-2920.2005.00816.x

Ma, B., Wang, H., Dsouza, M., Lou, J., He, Y., Dai, Z. et al. (2016). Geographic patterns of co-occurrence network topological features for soil microbiota at continental scale in eastern china. *ISME J.* 10, 1891-1901. doi: 10.1038/ismej.2015.261

Magoc, T., and Salzberg, S.L. (2011). Flash: Fast length adjustment of short reads to improve genome assemblies. *Bioinformatics* 27, 2957-2963. doi: 10.1093/bioinformatics/btr507

Olsen, S.R., and Sommers, L.E. (1982). Phosphorus. In: *Methods of soil analysis* (part 2). American Society of Agronomy Madison, USA, pp. 403–430.

Wang, Q., Garrity, G.M., Tiedje, J.M., and Cole, J.R. (2007). Naive bayesian classifier for rapid assignment of rrna sequences into the new bacterial taxonomy. *Appl. Environ. Microbiol.* 73, 5261-5267. doi: 10.1128/Aem.00062-07

Zhang, J., Liu, Y.-X., Zhang, N., Hu, B., Jin, T., Xu, H. et al. (2019). NRT1.1B is associated with root microbiota composition and nitrogen use in field-grown rice. *Nat. Biotechnol.* 37, 676-684. doi: 10.1038/s41587-019-0104-4

Zhao, Z.B., He, J.Z., Geisen, S., Han, L.L., Wang, J.T., Shen, J.P. et al. (2019). Protist communities are more sensitive to nitrogen fertilization than other microorganisms in diverse agricultural soils. *Microbiome* 7, 33. doi: 10.1186/s40168-019-0647-0
